# The magnitude and population burden of educational inequalities in adverse birth outcomes

**DOI:** 10.1038/s41598-026-37601-z

**Published:** 2026-02-11

**Authors:** Anton Schreuder, David van Klaveren, Richard M. K. van Dijk, Jasper V. Been, Lisa Broeders, Ageeth N. Rosman, Wessel Kraaij, Tanja A. J. Houweling

**Affiliations:** 1 Department of Public Health, Erasmus MC, University Medical Center Rotterdam, Rotterdam, 3000 CA Netherlands; 2 Leiden Institute of Advanced Computer Science, Leiden University, Leiden, the Netherlands; 3 Division of Neonatology, Department of Neonatal and Paediatric Intensive Care, Erasmus MC Sophia Children’s Hospital, University Medical Centre Rotterdam, Rotterdam, Netherlands; 4 Department of Obstetrics and Gynaecology, Erasmus MC Sophia Children’s Hospital, University Medical Centre Rotterdam, Rotterdam, Netherlands; 5 Perined, Utrecht, Netherlands; 6 Department of Health Innovations, Rotterdam University of Applied Sciences, Rotterdam, Netherlands; 7 Netherlands Organisation for Applied Scientific Research, Leiden, Netherlands

**Keywords:** Epidemiology, Parturition, Public health, Educational status, Health equity, Diseases, Health care, Medical research, Risk factors

## Abstract

We aimed to describe the magnitude and population burden of inequality in adverse birth outcomes by maternal education (five categories). Data from 639,007 singleton births of gestational age ≥ 24 weeks or, if missing, birthweight ≥ 500 g were included from the National registry of the Netherlands, 2016–2019. One in six Dutch births had an adverse outcome. Each step down the educational ladder was associated with higher adverse outcome rates. Inequalities in stillbirth and neonatal mortality rates were large between the highest (Master’s or higher) and lowest (primary) educated groups (rate ratio [RR] = 2.94 [95%CI: 2.33–3.55] and 2.25 [1.71–2.79], respectively). Inequalities were smaller for preterm birth, small-for-gestational-age, low Apgar score, neonatal intensive care unit admission, and severe congenital anomalies (RR range = 1.32–1.77; PAF range = 13.8–17.7%). Mortality would reduce by a third if the entire population had the mortality rates of the highest educated (population attributable fraction [PAF]_stillbirth_=35.0% [24.4–45.6%]; PAF_neonatal mortality_=27.1% [14.4–39.7%]). The middle education category (higher secondary) was the largest group, contributing most to the population burden of inequality despite only having a moderately elevated risk of adverse outcomes. Population health gains would be largest if addressed through preventive approaches targeting the entire health gradient rather than by only focussing on those at highest risk.

**Supplementary Information:**

The online version contains supplementary material available at 10.1038/s41598-026-37601-z.

## Introduction

Circumstances from as early as conception influence health and success later in life^1^. Adverse birth outcomes such as preterm birth or small-for-gestational-age (SGA) increase the risk of adverse health and social outcomes across the life course^2–5^. The risk of adverse birth outcomes is substantially higher among socioeconomically disadvantaged groups, even in welfare states^6^. These inequalities contribute to the intergenerational transmission of health and social (dis)advantage, resulting in a persistence of health^7^.

Quantification of both the magnitude and population-health burden of inequality in birth outcomes is important for supporting policies. Evidence on the population burden of inequality across different socioeconomic groups, for example, can inform whether strategies should take a targeted or population-wide approach.

Maternal education – one of the dimensions of socioeconomic position (SEP)—is a particularly important determinant of perinatal and child health, among others via behavioral pathways like smoking and through underlying structural factors associated with SEP^7,8^. Yet, evidence from nationally representative or nationwide data on educational inequalities in birth outcomes remains scant and restricted to a limited set of birth outcomes^9–15^ There is some evidence from birth cohort studies^16–18^, but these underrepresent lower socioeconomic groups^19^. In the Netherlands, evidence on socioeconomic inequalities in birth outcomes using nationwide data remains restricted to neighbourhood level measures of SEP^20–25^. Both unrepresentative samples and geographical measures of SEP lead to underestimation of the magnitude and burden of inequality^26^. To our knowledge, there is no nationwide study on the magnitude and burden of inequality by maternal education for a broad set of outcomes in the Netherlands. Our study hereby aimed to measure this using Dutch nationwide registration data from 2016 to 2019, i.e., the most recently available reliable national-level registration data.

## Methods

### Study population and data sources

Our study population consists of all births in the Netherlands between 1 January 2016 and 31 December 2019. Data from beyond 2019 were not included because of disruptions due to Covid. We used the following exclusion criteria: no legal mother registered, multiple birth, and gestational age < 24 weeks (in the Netherlands, active management is not provided for infants born before 24 weeks)^27,28^. If gestational age was missing, then missing birthweight or birthweight < 500 g were applied as additional exclusion criteria^28^.

Individual-level data were used from Statistics Netherlands, including birth data from the Netherlands Perinatal Registry (Perined; data request number 22.17), within a linked data-infrastructure known as DIAPER (Data-InfrAstructure for ParEnts and childRen)^29–31^. Perined works together with health care providers in the routine collection of perinatal data, covering approximately 97% of births in the Netherlands. Perined data were individually linked to data on maternal education and other background characteristics using the record identification number assigned by Statistics Netherlands (CBS), each of which represents a pseudonymized Dutch citizen. The study was conducted in accordance with the nationally and internationally accepted standards for scientific conduct as stated in the Netherlands Code of Conduct for Research Integrity (2018). The need to obtain the informed consent was waived by the Medical Ethics Committee (METC) of Erasmus MC, Rotterdam, The Netherlands, who concluded that the rules laid down in the Medical Research Involving Human Subjects Act do not apply to our study (MEC-2021-0846).

### Variables

**Fig. 1:**
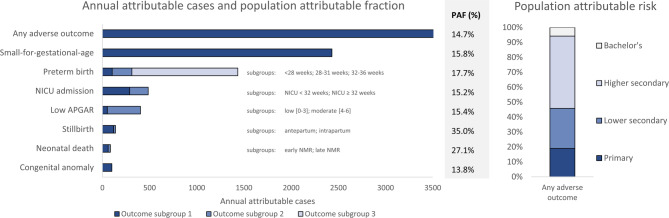
Population burden of inequalities in adverse birth outcomes. The left-hand graph gives the average crude PAF and annual crude reduction in the number of adverse birth outcomes for the scenario that all educational groups had the same birth outcome rates as the highest educational group. The right-hand graph gives the contribution of each of the educational groups to the population-level burden of inequality expressed as crude population attributable risk. NICU = neonatal intensive care unit; PAF = population attributable fraction.

We investigated seven adverse birth outcomes (definitions and subcategories in brackets): stillbirth (fetal death at ≥ 24weeks of gestation; subcategories: antepartum stillbirth, intrapartum stillbirth); neonatal mortality (death during the first 28 days of life; subcategories: early neonatal mortality [death on day 1–7], late neonatal mortality [death on day 8–28]); preterm birth (gestational age < 37 weeks [estimated date of conception is the first day of the last menstruation]; we used the following mutually exclusive subcategories: extremely preterm [gestational age < 28 weeks], very preterm [28–31 weeks], moderate/late preterm [32–36 weeks]; SGA (Hoftiezer percentile < 10^32^; Apgar score at 5 min < 7 (subcategories: low [0–3], moderate [4–6]^33^; neonatal intensive care unit (NICU) admission (subcategories: admission at gestational age < 32 weeks, admission at gestational age ≥ 32 weeks); and severe congenital anomalies (recorded postnatally by a health professional (List S1)). The following composite outcomes were defined: any adverse outcome (for all births and for live births only), and a Big 4 outcome (i.e., the four leading precursors of perinatal death in the Netherlands: preterm birth, SGA, five-minute Apgar < 7, and severe congenital anomaly) (Fig. 1).

Educational attainment was divided into five categories: primary education (ISCED 2011 levels 0–1, lower secondary education (ISCED level 2), higher secondary education (ISCED levels 3–4), bachelor’s degree (ISCED levels 5–6), and master’s degree or higher (ISCED levels 7–8) (see “Dutch educational levels” in Supplement)^34^.

### Statistical analyses

Descriptive statistics and outcome frequencies before and after imputations are given in Tables 1 and S2, respectively. We estimated adverse birth outcome rates for each maternal educational group by dividing the number of adverse outcomes by the number of (live) births. We estimated absolute and relative inequality in birth outcomes using the rate difference and the rate ratio (RR), respectively, of the lowest (primary education) and highest (Master’s degree or higher) education groups.

**Table 1:** Demographic characteristics and outcomes frequency.

Variable	Frequency (%)
Educational level, missing = 69,792 (10.9%)	
1 (Primary education)	24,233 (4.26)
2 (Lower secondary education)	47,933 (8.42)
3 (Higher secondary education)	229,275 (40.28)
4 (Bachelor’s degree)	162,213 (28.50)
5 (Master’s degree or higher)	105,561 (18.55)
Age, missing = 135 (0.02%)	
< 24 years	38,750 (6.07)
25–30 years	265,579 (41.57)
≥ 31 years	334,543 (52.36)
Parity, missing = 2848 (0.5%)	
0	278,750 (43.82)
1	230,349 (36.21)
≥ 2	127,060 (19.97)
Vital outcome, missing = 27 (< 0.01%)	
Antepartum stillbirths	1344 (0.21)
Intrapartum stillbirths	242 (0.04)
Early neonatal death	985 (0.15)
Late neonatal death	228 (0.04)
Gestational age, missing = 3662 (0.6%)	
Extremely preterm birth	1778 (0.28)
Very preterm birth	3448 (0.54)
Moderate preterm birth	27,987 (4.41)
Postterm birth	8667 (1.36)
Hoftiezer percentile, missing = 9014 (1.4%)	
0–10%	61,337 (9.74)
90–100%	64,274 (10.20)
5-minute Apgar score, missing = 1074 (0.2%)	
0–3	3313 (0.52)
4–6	8622 (1.35)
NICU admission, missing = 3662 (0.6%)	
Gestational age < 32 weeks	4126 (0.65)
Gestational age ≥ 32 weeks	8535 (1.34)

We estimated three measures of the population burden of inequality: the population attributable risk (PAR), the population attributable fraction (PAF), and the annual attributable cases^8^. The PAF and the PAR are now part of the repertoire of social epidemiology to measure the population burden of inequality. The PAR represents the absolute reduction in birth outcome rate for the scenario that the entire population had the same birth outcome rates as the highest educated. It is calculated by subtracting the rate of the highest education group from the population rate. The PAF represents the proportional reduction in adverse birth outcomes in the above scenario, and is equal to the PAR divided by the population rate. The number of annual attributable cases is the estimated yearly number of cases that would be avoided in this scenario, and is calculated as the yearly average difference between the actual number of cases and the estimated number of cases in the above scenario.

We used multiple imputation to assign probable values to missing data on maternal education, age, parity, and outcomes^35^. Five iterations were used for each imputed dataset, using predictive mean matching for numeric data, logistic regression imputation for dichotomous data, and polytomous regression imputation for categorical data. The covariates “partner’s educational level” and “same household” were not imputed due to their large proportion of missing values (6.9% and 18.7%, respectively), i.e., these covariates were not used as predictors in the multiple imputation when missing. Further details on how missing data were handled are provided in the supplement. Ten imputed datasets were created for pooling the estimates’ means and confidence intervals using Rubin’s rules^36^. The accuracy of multiple imputations for educational attainment – the variable with most missing values – was calculated by performing multiple imputations on a dataset of participants with known educational levels. This was done by assigning a percentage of the known educational levels as missing at random (equivalent to the actual proportion of missings) and reporting the proportion of correctly imputed values.

The PAR, PAF, and attributable cases were calculated with and without adjustment for age and parity to examine to what extent the burden of inequality in birth outcomes is explained by educational differences in reproductive behaviors^37^. For this analysis, age was divided into similarly sized quartiles (< 28 years, 28–30 years, 31–33 years, > 33 years) and parity was divided into three categories: 0, 1, and > 1. Summing the individual risk probabilities derived from fitted multivariable (multinomial) logistic regression models provides the expected number of adverse outcomes within each group after adjustment, with which other adjusted measures can be derived^38^.

Three sensitivity analyses were performed: (i) replacing attained educational level at birth with attained educational level five years after birth (only available for 2016–2017 birth cohort) to account for mothers who were still to complete their educational trajectory at time of birth; (ii) exclusion of births with missing data (unimputed dataset); and (iii) adjustment for all determinants described in the supplement (excluding partner’s educational level and “same household” due to the high proportion of missings for these variables).

All statistical analyses were performed in statistical program R version 4.1.3^39^.

## Results

Perined recorded 671,088 births between 2016 and 2019 in the Netherlands, of which 641,582 (95.6%) could be linked to Statistics Netherlands data (Figure S1). After applying the exclusion criteria, 639,007 (95.2%) births were included in our analyses. Information on maternal education was missing for 69,792 (10.9%) records. Missing information on birth outcomes was rare (Table 1).

The lower two educational groups were comparatively small, making up 4.3% and 8.4% of the population, whereas the middle group (higher secondary education) was substantial (40.3%; Table 1). Mothers with higher educational levels were more often older and had fewer births than lower-educated mothers.

Almost 16% of births had an adverse outcome, ranging from 13.5% in the highest education group to 21.1% in the lowest group (Table 2). The absolute number of adverse outcomes in the lowest education group represented only 7.5% of all adverse outcomes (Figure S2). Among liveborn infants, 15.1% (95% confidence interval = 15.0-15.2%) had a Big 4 outcome (preterm, SGA, 5 min Apgar < 7, or severe congenital anomaly). Nearly 15% of adverse birth outcomes (average of 3703 [3357–4049] yearly cases; 3583 [3239–3927] in liveborn infants) would be avoided if all groups had the same birth outcome rates as the highest education group. A gradient was observed for most outcomes, with systematically worse birth outcomes each step down the educational ladder (Tables 2, 3 and 4).

**Table 2:** Composite outcome and vital outcome rates stratified by maternal education.

Outcome	Composite outcomes			Vital outcomes					
Outcome (sub)group	Any adverse outcome, % births	Any adverse outcome, % live births	Big 4 outcome, % live births	Stillbirths, /1000 births	Antepartum stillbirths, /1000 births	Intrapartum stillbirths, /1000 births	Total neonatal deaths, /1000 live births	Early neonatal deaths, /1000 live births	Late neonatal deaths, /1000 live births
Total population rate	15.79 (15.70 to 15.88)	15.58 (15.49 to 15.67)	15.10 (15.01 to 15.19)	2.50 (2.38 to 2.62)	2.12 (2.01 to 2.23)	0.38 (0.33 to 0.43)	1.92 (1.81 to 2.03)	1.57 (1.47 to 1.67)	0.36 (0.31 to 0.41)
*Maternal education*									
Primary education, rate	21.07 (20.65 to 21.49)	20.69 (20.27 to 21.11)	20.15 (19.74 to 20.56)	4.78 (4.07 to 5.49)	4.00 (3.35 to 4.65)	0.78 (0.49 to 1.07)	3.16 (2.58 to 3.74)	2.68 (2.15 to 3.21)	0.47 (0.25 to 0.69)
Lower secondary, rate	20.31 (19.93 to 20.69)	20.02 (19.65 to 20.39)	19.50 (19.13 to 19.87)	3.64 (3.08 to 4.20)	3.07 (2.55 to 3.59)	0.57 (0.35 to 0.79)	2.29 (1.84 to 2.74)	1.72 (1.33 to 2.11)	0.57 (0.35 to 0.79)
Higher secondary, rate	16.46 (16.31 to 16.61)	16.25 (16.11 to 16.39)	15.78 (15.64 to 15.92)	2.53 (2.33 to 2.73)	2.15 (1.97 to 2.33)	0.38 (0.30 to 0.46)	2.19 (2.01 to 2.37)	1.75 (1.59 to 1.91)	0.44 (0.36 to 0.52)
Bachelor’s, rate	13.81 (13.65 to 13.97)	13.62 (13.46 to 13.78)	13.16 (13.00 to 13.32)	2.20 (1.98 to 2.42)	1.87 (1.67 to 2.07)	0.33 (0.25 to 0.41)	1.52 (1.34 to 1.70)	1.26 (1.09 to 1.43)	0.26 (0.19 to 0.33)
Master’s or higher, rate (ref.)	13.47 (13.27 to 13.67)	13.33 (13.13 to 13.53)	12.85 (12.66 to 13.04)	1.63 (1.40 to 1.86)	1.37 (1.16 to 1.58)	0.26 (0.17 to 0.35)	1.40 (1.19 to 1.61)	1.20 (1.00 to 1.40)	0.21 (0.13 to 0.29)
Rate difference	7.60 (7.14 to 8.06)	7.36 (6.90 to 7.82)	7.30 (6.84 to 7.76)	3.15 (2.40 to 3.90)	2.63 (1.94 to 3.32)	0.52 (0.22 to 0.82)	1.75 (1.13 to 2.37)	1.48 (0.91 to 2.05)	0.27 (0.03 to 0.51)
Rate ratio	1.56 (1.52 to 1.60)	1.55 (1.51 to 1.59)	1.57 (1.53 to 1.61)	2.94 (2.33 to 3.55)	2.93 (2.27 to 3.59)	3.02 (1.47 to 4.57)	2.25 (1.71 to 2.79)	2.24 (1.66 to 2.82)	2.30 (0.87 to 3.73)
Crude results									
PAR	2.32 (2.10 to 2.54)	2.25 (2.03 to 2.47)	2.25 (2.04 to 2.46)	0.88 (0.62 to 1.14)	0.75 (0.51 to 0.99)	0.12 (0.02 to 0.22)	0.52 (0.28 to 0.76)	0.37 (0.15 to 0.59)	0.15 (0.05 to 0.25)
PAF, %	14.7 (13.3 to 16.0)	14.4 (13.0 to 15.8)	14.9 (13.5 to 16.3)	35.0 (24.4 to 45.6)	35.5 (23.9 to 47.0)	32.4 (3.9 to 61.0)	27.1 (14.4 to 39.7)	23.5 (9.3 to 37.7)	42.5 (14.8 to 70.1)
Annual attributable cases	3703 (3357 to 4049)	3583 (3239 to 3927)	3586 (3247 to 3925)	140 (97 to 183)	120 (80 to 160)	20 (2 to 38)	83 (44 to 122)	59 (23 to 95)	24 (8 to 40)
Adjusted results									
PAR	3.01 (2.79 to 3.23)	2.93 (2.71 to 3.15)	2.91 (2.70 to 3.12)	0.96 (0.70 to 1.22)	0.82 (0.58 to 1.06)	0.14 (0.04 to 0.24)	0.55 (0.31 to 0.79)	0.38 (0.16 to 0.60)	0.17 (0.07 to 0.27)
PAF, %	19.1 (17.7 to 20.4)	18.8 (17.4 to 20.2)	19.3 (17.9 to 20.7)	38.3 (27.6 to 48.9)	38.5 (27.0 to 50.0)	37.0 (9.0 to 65.0)	28.6 (16.0 to 41.2)	24.6 (10.1 to 39.0)	46.2 (19.5 to 72.9)
Annual attributable cases	4808 (4461 to 5155)	4675 (4329 to 5020)	4643 (4303 to 4983)	153 (110 to 196)	130 (90 to 170)	23 (5 to 41)	88 (49 to 127)	61 (25 to 97)	26 (11 to 41)

The values provided are pooled means across all imputed datasets, with the pooled 95% confidence intervals given in brackets. Rate difference: rate in lowest education group minus rate in highest education group. Rate ratio: rate in lowest education group divided by rate in highest education group. The Big 4 outcomes are preterm birth, small-for-gestational-age, Apgar score at 5 min < 7, and severe congenital anomaly. PAF = population attributable fraction; PAR = population attributable risk.

**Table 4:** Apgar score at 5 min, NICU admission, and severe congenital anomaly rates stratified by maternal education.

Outcome	5-minute Apgar score			NICU admissions			Severe congenital anomaly
Outcome (sub)group	Total Apgar scores < 7, %	Apgar scores 0–3, %	Apgar scores 4–6, %	Total NICU admissions, %	< 32 weeks NICU admissions, %	≥ 32 weeks NICU admission, %	Severe congenital anomaly, /1000
Total population rate	1.63 (1.60 to 1.66)	0.27 (0.26 to 0.28)	1.36 (1.33 to 1.39)	2.00 (1.97 to 2.03)	0.65 (0.63 to 0.67)	1.35 (1.32 to 1.38)	4.54 (4.37 to 4.71)
Maternal education							
Primary education, rate	2.22 (2.07 to 2.37)	0.38 (0.32 to 0.44)	1.84 (1.70 to 1.98)	2.47 (2.31 to 2.63)	0.77 (0.68 to 0.86)	1.70 (1.57 to 1.83)	6.81 (5.96 to 7.66)
Lower secondary, rate	1.85 (1.72 to 1.98)	0.29 (0.24 to 0.34)	1.56 (1.44 to 1.68)	2.44 (2.30 to 2.58)	0.87 (0.78 to 0.96)	1.57 (1.45 to 1.69)	4.75 (4.11 to 5.39)
Higher secondary, rate	1.69 (1.64 to 1.74)	0.28 (0.26 to 0.30)	1.41 (1.36 to 1.46)	2.12 (2.06 to 2.18)	0.74 (0.71 to 0.77)	1.38 (1.33 to 1.43)	4.66 (4.39 to 4.93)
Bachelor’s, rate	1.51 (1.45 to 1.57)	0.25 (0.23 to 0.27)	1.26 (1.21 to 1.31)	1.79 (1.73 to 1.85)	0.55 (0.52 to 0.58)	1.24 (1.19 to 1.29)	4.25 (3.95 to 4.55)
Master’s or higher, rate (ref.)	1.38 (1.31 to 1.45)	0.24 (0.21 to 0.27)	1.13 (1.07 to 1.19)	1.70 (1.63 to 1.77)	0.47 (0.43 to 0.51)	1.23 (1.17 to 1.29)	3.91 (3.55 to 4.27)
Rate difference	0.84 (0.67 to 1.01)	0.14 (0.07 to 0.21)	0.70 (0.55 to 0.85)	0.77 (0.59 to 0.95)	0.30 (0.20 to 0.40)	0.48 (0.33 to 0.63)	2.90 (1.98 to 3.82)
Rate ratio	1.61 (1.47 to 1.75)	1.57 (1.25 to 1.89)	1.62 (1.47 to 1.77)	1.46 (1.35 to 1.57)	1.63 (1.40 to 1.86)	1.39 (1.26 to 1.52)	1.74 (1.47 to 2.01)
Crude results							
PAR	0.25 (0.18 to 0.32)	0.03 (0.00 to 0.06)	0.22 (0.15 to 0.29)	0.30 (0.22 to 0.38)	0.18 (0.14 to 0.22)	0.12 (0.05 to 0.19)	0.63 (0.23 to 1.03)
PAF, %	15.4 (10.9 to 20.0)	11.3 (-0.4 to 23.0)	16.3 (11.3 to 21.3)	15.2 (11.0 to 19.4)	27.8 (20.9 to 34.7)	9.1 (3.8 to 14.4)	13.8 (5.1 to 22.5)
Annual attributable cases	401 (282 to 520)	49 (-2 to 100)	351 (243 to 459)	484 (351 to 617)	289 (217 to 361)	196 (82 to 310)	100 (37 to 163)
Adjusted results							
PAR	0.35 (0.28 to 0.42)	0.05 (0.02 to 0.08)	0.31 (0.24 to 0.38)	0.41 (0.33 to 0.49)	0.20 (0.16 to 0.24)	0.21 (0.14 to 0.28)	0.81 (0.41 to 1.21)
PAF, %	21.8 (17.2 to 26.4)	17.8 (6.8 to 28.8)	22.6 (17.7 to 27.5)	20.6 (16.4 to 24.7)	31.2 (24.3 to 38.2)	15.4 (10.3 to 20.5)	17.9 (9.2 to 26.7)
Annual attributable cases	566 (445 to 687)	77 (29 to 125)	488 (381 to 595)	655 (523 to 787)	324 (251 to 397)	331 (221 to 441)	130 (66 to 194)

The values provided are pooled means across all imputed datasets, with the pooled 95% confidence interval given in brackets. Rate difference: rate in lowest education group minus rate in highest education group. Rate ratio: rate in lowest education group divided by rate in highest education group. All rates are reported as the proportion of live births. Adjusted results are adjusted for age and parity. NICU = neonatal intensive care unit; PAF = population attributable fraction; PAR = population attributable risk.

Relative inequalities in vital outcomes were large (Table 2). The stillbirth rate was nearly three times as high in the lowest compared with the highest education group (RR = 2.94, 95% confidence interval = 2.33–3.55). While most stillbirths occurred before start of birth, a systematic gradient and large relative inequalities were also observed for intrapartum stillbirths (RR = 3.02, 1.47–4.57). The neonatal mortality rate was more than twice as high among the lowest compared with the highest education group (RR = 2.25, 1.71–2.79). Neonatal mortality was higher in the early compared to the late neonatal phase; the RR was similar for both subgroups. Mortality rates would reduce by around one-third if the entire population had the mortality rates observed in the highest education group (PAF_stillbirth rate_: 35.0%, 24.4–45.6%; PAF_neonatal mortality rate_: 27.1%, 14.4–39.7%). In absolute terms, the stillbirth rate in the total population would be reduced by 0.88 (0.62–1.14) per 1000 births and the neonatal mortality rate by 0.52 (0.28–0.76) per 1000 live births if all had the mortality rate of the highest education group, representing an average of 140 (97–183) stillbirths and 83 (44–122) neonatal deaths per year.

5% ﻿of liveborn infants were born prematurely (95% confidence interval = 5.0-5.1%), consisting mostly of moderate/late preterm births (Table 3). Inequalities were modest overall (RR = 1.32, 1.25–1.39), and RRs increased with shorter gestational age. The relative burden of inequality in prematurity was moderate (PAF = 17.7%, 15.2–20.2%). Yet, as prematurity applied to a greater proportion of infants than the vital outcomes, the number of yearly attributable cases was comparatively large (*n* = 1433, 1229–1637).

**Table 3:** Preterm birth and small-for-gestational-age rates stratified by maternal education.

Outcome	Preterm births				Small-for-gestational age
Outcome (sub)group	Total PTBs (< 37 weeks), %	Extremely PTBs (< 28 weeks), %	Very PTBs (28–32 weeks), %	Moderate/late PTBs (33–36 weeks), %	Hoftiezer percentile < 10, %
Total population rate	5.09 (5.04 to 5.14)	0.22 (0.21 to 0.23)	0.51 (0.49 to 0.53)	4.36 (4.31 to 4.41)	9.67 (9.60 to 9.74)
Maternal education					
Primary education, rate	5.52 (5.28 to 5.76)	0.27 (0.22 to 0.32)	0.60 (0.52 to 0.68)	4.64 (4.42 to 4.86)	14.42 (14.06 to 14.78)
Lower secondary, rate	6.40 (6.17 to 6.63)	0.31 (0.26 to 0.36)	0.65 (0.58 to 0.72)	5.43 (5.22 to 5.64)	13.28 (12.96 to 13.60)
Higher secondary, rate	5.47 (5.38 to 5.56)	0.26 (0.24 to 0.28)	0.57 (0.54 to 0.60)	4.64 (4.56 to 4.72)	10.03 (9.91 to 10.15)
Bachelor’s, rate	4.62 (4.52 to 4.72)	0.17 (0.15 to 0.19)	0.44 (0.41 to 0.47)	4.00 (3.91 to 4.09)	8.03 (7.90 to 8.16)
Master’s or higher, rate (ref.)	4.19 (4.07 to 4.31)	0.16 (0.14 to 0.18)	0.38 (0.34 to 0.42)	3.65 (3.54 to 3.76)	8.15 (7.99 to 8.31)
Rate difference	1.33 (1.07 to 1.59)	0.12 (0.06 to 0.18)	0.22 (0.13 to 0.31)	0.99 (0.75 to 1.23)	6.27 (5.87 to 6.67)
Rate ratio	1.32 (1.25 to 1.39)	1.75 (1.32 to 2.18)	1.58 (1.32 to 1.84)	1.27 (1.20 to 1.34)	1.77 (1.71 to 1.83)
Crude results					
PAR	0.90 (0.77 to 1.03)	0.06 (0.03 to 0.09)	0.13 (0.09 to 0.17)	0.70 (0.58 to 0.82)	1.53 (1.36 to 1.70)
PAF, %	17.7 (15.2 to 20.2)	29.1 (16.5 to 41.7)	25.6 (17.8 to 33.4)	16.2 (13.4 to 18.9)	15.8 (14.0 to 17.6)
Annual attributable cases	1433 (1229 to 1637)	102 (58 to 146)	208 (144 to 272)	1122 (930 to 1314)	2431 (2154 to 2708)
Adjusted results					
PAR	1.06 (0.93 to 1.19)	0.07 (0.04 to 0.10)	0.15 (0.11 to 0.19)	0.84 (0.72 to 0.96)	2.01 (1.84 to 2.18)
PAF, %	20.8 (18.3 to 23.3)	30.1 (18.9 to 41.4)	30.0 (22.0 to 38.0)	19.2 (16.5 to 21.9)	20.8 (19.0 to 22.6)
Annual attributable cases	1682 (1478 to 1886)	106 (66 to 146)	244 (179 to 309)	1333 (1143 to 1523)	3199 (2921 to 3477)

The values provided are pooled means across all imputed datasets, with the pooled 95% confidence intervals given in brackets. Rate difference: rate in lowest education group minus rate in highest education group. Rate ratio: rate in lowest education group divided by rate in highest education group. All rates are reported as the proportion of live births. Adjusted results are adjusted for age and parity. PAF = population attributable fraction; PAR = population attributable risk; PTB = preterm birth.

A similar pattern was found for infants born SGA (Table 3). The percentage of infants born SGA was 1.77 times higher in the lowest compared with the highest education group (95% confidence interval = 1.71–1.83). 15.8% (14.0-17.6%) of SGA cases were attributable to inequality in SGA rates between educational groups, representing 2431 (2154–2708) yearly attributable cases.

1.63% of live born infants had a low Apgar score (95% confidence interval = 1.60–1.66%), and 2.00% (1.97–2.03%) were admitted to the NICU (Table 4). These mostly consisted of less severe cases, namely Apgar scores 4 to 6 and cases admitted to NICU after 32 weeks’ gestational age. The PAF was 15.4% (10.9–20.0%) for low Apgar score and 15.2% (11.0-19.4%) for NICU admission, equivalent to an average of 401 (282–520) and 484 (351–617) adjusted attributable cases per year, respectively.

Finally, almost 5 per 1000 live born infants had a severe congenital anomaly (95% confidence interval = 4.37–4.71%), with rates ranging from 3.91 (3.55–4.27) per 1000 in the highest education group to 6.81 (5.96–7.66) per 1000 in the lowest education group (Table 4). Severe congenital anomalies would be reduced by 13.8% (5.1–22.5%) if all had the rate of the highest education group, and 100 cases (37–163) would be avoided yearly.

Inequalities in adverse birth outcomes were larger after adjustment for differences in reproductive behavior between lower and higher educated women, the latter being of more advanced age and more often primiparous. Hence, also the PAF, PAR, and number of attributable cases increased after adjustment for age and parity (Tables 2, 3 and 4,S3-S11). When adjusted for all covariates, the PAF decreased somewhat (e.g., from 14.7% to 13.2% for any adverse birth outcome); only for neonatal mortality was the decline substantial (from 27.1% to 15.8%) (Tables S9-S11).

Sensitivity analyses using maternal educational level attained at five years in the future instead of education at birth provided similar results to using educational level at the time of birth (Tables S3–S5). The unimputed dataset had lower population rates for all birth outcomes compared to the imputed datasets, but otherwise showed similar results (Tables S6–S8).

## Discussion

### Main findings

Using the most recently available reliable nationwide population registry data, our study shows that educational inequalities in adverse birth outcomes pervade the entire Dutch society. These adverse outcomes are not just a problem of the most deprived. Rather, there is a systematic gradient, with each step down the socioeconomic ladder being associated with worse outcomes.

Furthermore, although the rates of adverse birth outcomes were highest among the lowest educated, the number of births with an adverse outcome was small in this group. The middle education group—with moderately elevated rates of adverse outcomes—contributed most to the population burden of inequality. This aligns with the prevention paradox: most cases of death or illness occur in the low- to moderate-risk group, while only a minority occur in the high-risk group^40^.

Reducing inequalities in adverse birth outcomes across the entire gradient would lead to considerable population health gains. If all population groups had the mortality rates of the highest educated, population-level stillbirth and neonatal mortality rates would reduce by approximately 30%. As these vital outcomes are rare, the absolute number of these severe outcomes avoided would remain modest. Conversely, the number of cases avoided would be substantial for more prevalent problems like SGA and preterm birth. Reproductive behaviors among higher educated women—namely fewer births and births at a more advanced age – had a tempering effect on inequality, as these behaviors are associated with higher risk of adverse birth outcomes^37^.

Our study is the first to estimate the magnitude of inequality by maternal education for a comprehensive set of birth outcomes using routinely collected Dutch nationwide data. Furthermore, our study adds to the literature by quantifying the population burden of inequality in these outcomes and the contribution of different socioeconomic groups to this burden. Our paper hereby illustrates how information on RR and group size can be used to generate evidence that is relevant for policy making and monitoring of inequalities.

### Interpretation

What explains these socioeconomic inequalities in birth outcomes? Proximal determinants of birth outcomes revolve around physical and mental health of the mother and health behaviors before and during pregnancy. This includes medical history, weight status, diet, exercise, stress, and exposure to toxic substances such as cigarette smoke^41–44^. Women in lower education groups are more likely to smoke and be exposed to passive smoking, be overweight/obese and experience unhealthy weight gain during pregnancy, suffer from gestational hypertension and mental health problems, and be less likely to take folic acid supplementation^17,45–51^. Maternal smoking explained two-thirds of educational inequalities in SGA in a Dutch birth cohort study^17^. Future research could usefully examine the relative contribution of such proximal determinants to inequalities in birth outcomes, for example using causal mediation analysis^52^.

Socioeconomic inequalities in maternal health and health behaviors are associated with structural inequalities in the conditions in which (expectant) families live, and the structural drivers of these conditions^1^. Maternal education, as one of the dimensions of socioeconomic position, is associated with income and occupational status. Poverty, low housing quality, job insecurity, and neighbourhood unsafety and deprivation contribute to inequalities in maternal health and health behaviors. In our analysis, inequalities in birth outcomes were partly explained by factors that are strongly associated with the material conditions in which families live such as household income, occupational status, and insurance debt. Yet, inequalities in birth outcomes by maternal education remained substantial even after adjustment for these covariates. This suggests that the social conditions in which families live play a role, including health behaviors in the social environment. The structural drivers of these material and social conditions include the way economies are structured, with lower income levels for jobs requiring lower education levels, social security, and employment policies (e.g., minimum wage, social benefits, housing policies, and urban planning), and the extent of social segregation^1,7^.

Inequalities in conditions in which families live and their structural drivers contribute to the intergenerational transmission of (dis)advantage. Such transmission operates through biological and social pathways. For example, SEP of the grandparents influences a mother’s birthweight and growth trajectory, both of which are independent predictors of her children’s birthweight^53^. Similarly, grandparental SEP influences maternal educational attainment, health behaviors, and health outcomes^54^. In other words, determinants of educational inequalities overlap with those of inequalities in birth outcomes.

The implications of adverse birth outcomes across the life-course, for cognitive and social-emotional development, and for educational attainment, and mental and physical health are substantial, and inequalities in adverse birth outcomes contribute to the intergenerational transmission of (dis)advantage^55^. Moreover, adverse birth outcomes affect parental mental health^56,57^. Reducing inequalities in adverse birth outcomes requires a long-term strategy given the intergenerational mechanisms at play.

A more structural approach is arguably needed to address the gradient in adverse birth outcomes. An approach that includes, but stretches beyond, those at highest risk by addressing the social determinants of health that underlie the gradient in health and health behaviors^1^. In other words, improving the conditions in which people are born, grow, live, and work, with proportionally more attention for those at higher risk of adverse birth outcomes. This includes, for example, reducing poverty and problematic debts, more stringent tobacco control laws, reducing air pollution, and urban planning that promotes healthy living and safe neighbourhoods^58,59^.

### Strengths and limitations

Some limitations should be considered. First, over 4% of all births could not be linked to Statistics Netherlands data. These linkage failures are likely due to the lack of a maternal Dutch identification number (e.g., tourists, asylum seekers). Notably, adverse birth outcomes in the unlinked cohort occurred at a higher rate than in the linked cohort, suggesting that our reported measures of inequality are underestimates. Conversely, the observed inequalities in severe congenital anomalies may be partially explained by differential elective termination of pregnancy in relation to such anomalies. Unfortunately, no reliable data on termination were available to investigate this.

Another limitation is that 10.9% of records missed information on maternal education. The accuracy of our missing values estimates using multiple imputation was 59.7% (Table S1), which is greater than 40.3% accuracy when assigning the majority class to all missing values (education level 3). Given that educational attainment is more likely to be missing for lower educated persons, inaccuracies in the imputation of missing values of educational attainment likely led to an underestimation of inequalities in birth outcomes. Finally, it is worth noting that causal estimation of the effects of maternal education on birth outcomes was beyond the scope of this paper.

## Conclusions

Socioeconomic inequalities in adverse outcomes are substantial and pervade the entire Dutch society, despite universal health insurance coverage. The burden of these inequalities in the population is large: a substantial number of adverse outcomes would be avoided if these inequalities were reduced. Population health gains would be largest if inequalities were addressed through approaches that address the entire health gradient rather than only by focussing on those at highest risk.

## Supplementary Information

Below is the link to the electronic supplementary material.

Supplementary Material 1

## Data Availability

The data that has been used is confidential. Access to the DIAPER linked data-infrastructure can be requested by contacting [diaper@rivm.nl] , access to Perined data can be requested by contacting [info@perined.nl] , and access to the CBS microdata can be requested by following the instructions at [https://www.cbs.nl/en-gb/our-services/customised-services-microdata/microdata-conducting-your-own-research/applying-for-access-to-microdata] .
