# Supplementary material for: The magnitude and population burden of educational inequalities in adverse birth outcomes
Source: Sci Rep. 2026 Feb 11;16:8280. doi: 10.1038/s41598-026-37601-z (PMC12966303; doi:10.1038/s41598-026-37601-z)
Supplement: Supplementary file 1 — Supplementary Material 1 [file 41598_2026_37601_MOESM1_ESM.docx]

# Supplement

**The magnitude and population burden of educational inequalities in adverse birth outcomes**

Anton Schreuder, David van Klaveren, Richard M.K. van Dijk, Jasper V. Been, Lisa Broeders, Ageeth N. Rosman, Wessel Kraaij, Tanja A.J. Houweling

## Contents

Contents

[Contents 2](#_Toc209979891)

[Dutch educational levels 3](#_Toc209979892)

[Handling missing data 3](#_Toc209979893)

### Dutch educational levels

Educational level is categorized by Statistics Netherlands into 18 ordinal levels according to the Dutch Standard Classification of Education (SOI). For years 2016 to 2018, this is according to the SOI 2016; for year 2019, this is according to SOI 2021. From lowest educational level to highest, these are (using original Dutch terms and abbreviations): [1] “Basisonderwijs groep 1-2”, [2] “Basisonderwijs groep 3-8”, [3] “Praktijkonderwijs”, [4] “Vmbo-b/k”, [5] “Mbo1”, [6] “Vmbo-g/t”, [7] “Havo-, vwo-onderbouw”, [8] “Mbo2”, [9] “Mbo3”, [10] “Mbo4”, [11] “Havo-bovenbouw”, [12] “Vwo-bovenbouw”, [13] “Hbo-associate degree”, [14] “Hbo-bachelor”, [15] “Wo-bachelor”, [16] “Hbo-master”, [17] “Wo-master”, [18] “Doctor”. The aggregation of these 18 levels into the 5 levels used for this study was performed in the following manner (with the International Standard Classification of Education (ISCED) 2011 levels given in brackets):

1. “Basisonderwijs” (primary education) = SOI levels 1-2 (ISCED 2011 levels 0-1),
2. “Vmbo, havo-,vwo-onderbouw, mbo1” (lower secondary education) = SOI levels 3-7 (ISCED Lower secondary education),
3. “Havo, vwo, mbo” (higher secondary education) = SOI levels 8-12 (ISCED levels 3-4),
4. “Hbo-, wo-bachelor” (bachelor’s degree) = SOI levels 13-15 (ISCED levels 5-6),
5. “Hbo-, wo-master, doctor” (master’s degree or higher) = SOI levels 16-18 (ISCED levels 7-8).

More detailed information is described here

<https://www.cbs.nl/nl-nl/onze-diensten/methoden/begrippen/onderwijsniveau-soi-2016>

and here

<https://www.cbs.nl/nl-nl/onze-diensten/methoden/begrippen/onderwijsniveau-soi-2021>.

### Handling missing data

Missing information on maternal education was imputed using covariables from Statistics Netherlands that have previously been demonstrated to be predictive of educational level (59). In addition to the adverse birth outcomes, the following covariables with respect to the mother were used: age (<24 years, 24-30 years, >30 years), parity (0, 1, >1), year (2016, 2017, 2018, 2019), Dutch province (Drenthe, Gelderland, Groningen, Flevoland, Friesland, Limburg, Noord-Brabant, Noord-Holland, Overijssel, Utrecht, Zeeland, Zuid-Holland), country of birth (Netherlands, other Europe, Turkey, Morocco, Suriname, Dutch Caribbean, Indonesia, other Africa, other Asia, other America and Oceania), parental country of birth (both parents born in the Netherlands, one parent born in the Netherlands, neither parent born in the Netherlands), at least six months’ health insurance debt in current or prior year (yes, no), criminal suspect in current or prior year (yes, no), occupational status (employee, director/majority shareholder, independent entrepreneur, other independent worker, receiving unemployment benefits, receiving social assistance benefits, receiving other social service benefits, receiving sickness benefits, receiving pension, school/university student with income, school/university student without income, other without income, cooperating family member). In addition, we used household income percentile (≤20%, 21%-40%, 41%-60%, 61%-80%, ≥81%, private household with unknown income, institutional household, private student household), partner's educational level (1-5), and living in same household with partner (yes, no) – subsequently referred to as “same household.” Note that the term “father” represents the mother's partner; this person may not be the biological parent and could be female.

### List S1: Severe congenital anomalies included in the study

- Anencephaly
- Meningomyelocele
- Hydranencephaly
- Congenital hydrocephalus
- Holoprosencephaly
- Gyration disorders/polymicrogyria
- Other lethal or life-threatening central nervous system abnormalities
- Transposition of the great vessels
- Tetralogy of Fallot (with and without pulmonary atresia)
- Tricuspid atresia
- Hypoplastic left heart syndrome
- Complex cor vitium
- Other lethal or life-threatening congenital heart defect
- Palatoschisis
- Colon or rectal atresia
- Anus imperforatus/anorectal malformation
- Omphalocele
- Gastroschisis
- Biliary atresia
- Small intestinal atresia
- Esophageal atresia (with or without fistula)
- Other lethal or life-threatening condition of the digestive tract
- Bilateral renal agenesis
- Bilateral polycystic, multicystic, or dysplastic kidneys
- Obstructive uropathy with congenital hydronephrosis
- Bladder exstrophy
- Other lethal or life-threatening disorder of the urogenital tract
- Trisomy 13
- Trisomy 18
- Trisomy 21
- Syndromic abnormalities (severe retrognathia, Treacher Collins, CHARGE, etc.)
- Other chromosomal abnormalities
- Skeletal dysplasia
- Congenital diaphragmatic hernia
- Hydrops fetalis
- Oligohydramnios sequence with the following features: (1) Oligohydramnios determined by ultrasound 5 or more days antepartum, (2) evidence of fetal movement restriction in postnatal physical
- Dystrophia myotonica
- conjoined twins
- Tracheal resia
- CCAML (Congenital cystic adenomatoid malformation of the lung)/CPAM (Congenital pulmonary airway malformations)
- Choaneatresia
- Other lethal or life-threatening respiratory illness
- Other lethal or life-threatening congenital anomalies


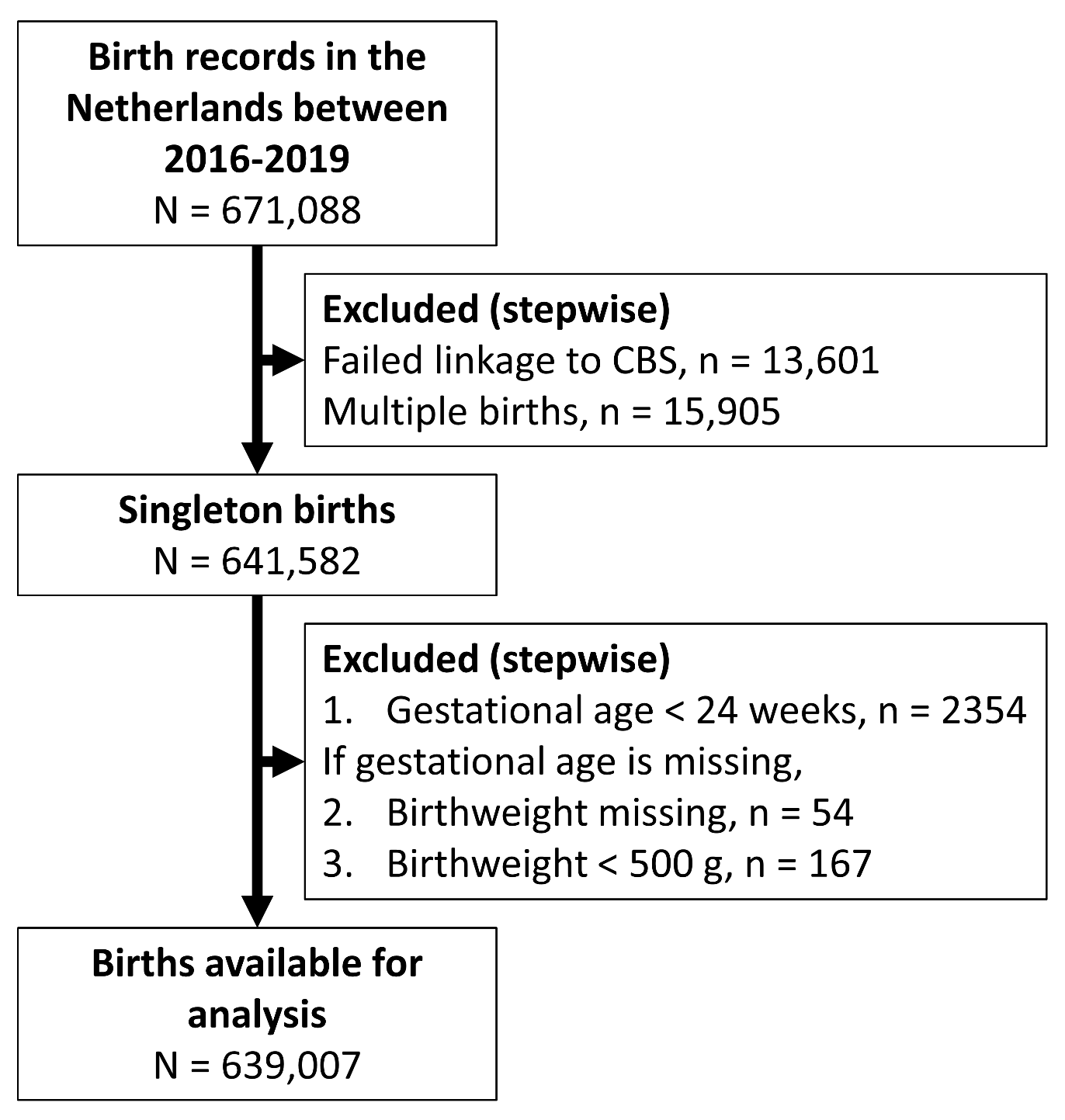


### Figure S1: Participant inclusion flowchart

### Figure S2: Number of births with an adverse outcome, by maternal highest attained educational level

### Table S1: Contingency table of multiple imputations accuracy for estimating maternal highest attained educational level in 10.9% of the known population

| True attained education  \  Estimated attained education | Primary education, n = 2639 | Lower secondary education, n = 5723 | Higher secondary education, n = 21369 | Bachelor’s degree, n = 17152 | Master’s degree or higher, n = 8613 |
| --- | --- | --- | --- | --- | --- |
| Primary education, n = 2785 | 1422 (51.1) | 559 (20.1) | 555 (19.9) | 172 (6.2) | 77 (2.8) |
| Lower secondary education, n = 4884 | 389 (8) | 2231 (45.7) | 1587 (32.5) | 515 (10.5) | 162 (3.3) |
| Higher secondary education, n = 22810 | 586 (2.6) | 2169 (9.5) | 14641 (64.2) | 4237 (18.6) | 1177 (5.2) |
| Bachelor’s degree, n = 15637 | 160 (1) | 530 (3.4) | 3322 (21.2) | 9619 (61.5) | 2006 (12.8) |
| Master’s degree or higher, n = 9380 | 82 (0.9) | 234 (2.5) | 1264 (13.5) | 2609 (27.8) | 5191 (55.3) |

The values given are the frequencies with column percentages given in brackets. The values in the left diagonal boxes were correctly estimated. The values in brackets are row percentages. Accuracy (correctly estimated values / total population) = 59.7%.

### Table S2: Covariable demographic characteristics and outcomes for one imputed dataset stratified by maternal education

| Variable | Total population,  n = 639,007 | Primary education,  n = 35981 (5.6%),  imputed = 11748 (32.7%) | Lower secondary education,  n = 58298 (9.1%),  imputed = 10365 (17.8%) | Higher secondary education,  n = 250914 (39.3%),  imputed = 21639 (8.6%) | Bachelor’s degree,  n = 177534 (27.8%),  imputed = 15,321 (8.6%) | Master’s or higher,  n = 116280 (18.2%),  imputed = 10719 (9.2%) |
| --- | --- | --- | --- | --- | --- | --- |
| Year |  |  |  |  |  |  |
| 2016 | 163339 (25.6) | 9543 (26.5) | 15676 (26.9) | 63748 (25.4) | 44997 (25.3) | 29375 (25.3) |
| 2017 | 160405 (25.1) | 9055 (25.2) | 14932 (25.6) | 63171 (25.2) | 44469 (25.0) | 28778 (24.7) |
| 2018 | 156623 (24.5) | 8960 (24.9) | 14109 (24.2) | 61268 (24.4) | 43524 (24.5) | 28762 (24.7) |
| 2019 | 158640 (24.8) | 8423 (23.4) | 13581 (23.3) | 62727 (25.0) | 44544 (25.1) | 29365 (25.3) |
| Age,  missing = 135 (0.02%) |  |  |  |  |  |  |
| ≤24 years | 38758 (6.1) | 5727 (15.9) | 11254 (19.3) | 20021 (8.0) | 1526 (0.9) | 230 (0.2) |
| 25-30 years | 265624 (41.6) | 12001 (33.4) | 24264 (41.6) | 132903 (53.0) | 69050 (38.9) | 27406 (23.6) |
| ≥31 years | 334625 (52.4) | 18253 (50.7) | 22780 (39.1) | 97990 (39.1) | 106958 (60.2) | 88644 (76.2) |
| Parity,  missing = 2848 (0.4%) |  |  |  |  |  |  |
| 0 | 280103 (43.8) | 10841 (30.1) | 22398 (38.4) | 112095 (44.7) | 80507 (45.3) | 54262 (46.7) |
| 1 | 231307 (36.2) | 10126 (28.1) | 19286 (33.1) | 90430 (36.0) | 67454 (38.0) | 44011 (37.8) |
| ≥2 | 127597 (20.0) | 15014 (41.7) | 16614 (28.5) | 48389 (19.3) | 29573 (16.7) | 18007 (15.5) |
| Province,  imputed = 28484 (4.5%) |  |  |  |  |  |  |
| Drenthe | 16387 (2.6) | 766 (2.1) | 1443 (2.5) | 7607 (3.0) | 4670 (2.6) | 1901 (1.6) |
| Flevoland | 18579 (2.9) | 1131 (3.1) | 2257 (3.9) | 9032 (3.6) | 4636 (2.6) | 1523 (1.3) |
| Friesland | 21244 (3.3) | 913 (2.5) | 1759 (3.0) | 9798 (3.9) | 6605 (3.7) | 2169 (1.9) |
| Gelderland | 76321 (11.9) | 3405 (9.5) | 6529 (11.2) | 32896 (13.1) | 21552 (12.1) | 11939 (10.3) |
| Groningen | 13586 (2.1) | 782 (2.2) | 1208 (2.1) | 5159 (2.1) | 3625 (2.0) | 2812 (2.4) |
| Limburg | 33715 (5.3) | 2170 (6.0) | 3575 (6.1) | 13804 (5.5) | 9174 (5.2) | 4992 (4.3) |
| Noord Brabant | 83502 (13.1) | 4925 (13.7) | 7690 (13.2) | 31903 (12.7) | 24770 (14.0) | 14214 (12.2) |
| Noord Holland | 110913 (17.4) | 6571 (18.3) | 9417 (16.2) | 37039 (14.8) | 30406 (17.1) | 27480 (23.6) |
| Overijssel | 45221 (7.1) | 1680 (4.7) | 3463 (5.9) | 19695 (7.8) | 14241 (8.0) | 6142 (5.3) |
| Utrecht | 56470 (8.8) | 2415 (6.7) | 4088 (7.0) | 17744 (7.1) | 16013 (9.0) | 16210 (13.9) |
| Zeeland | 12552 (2.0) | 651 (1.8) | 1356 (2.3) | 6290 (2.5) | 3247 (1.8) | 1008 (0.9) |
| Zuid Holland | 150517 (23.6) | 10572 (29.4) | 15513 (26.6) | 59947 (23.9) | 38595 (21.7) | 25890 (22.3) |
| Mother’s migration background,  imputed = 22 (<0.01%) |  |  |  |  |  |  |
| Dutch with one foreign parent | 124869 (19.5) | 28618 (79.5) | 18533 (31.8) | 39760 (15.8) | 20419 (11.5) | 17539 (15.1) |
| Born in other country | 79186 (12.4) | 1795 (5.0) | 8888 (15.2) | 36834 (14.7) | 19852 (11.2) | 11817 (10.2) |
| Mother’s parents’ nationalities,  imputed = 22 (<0.01%) |  |  |  |  |  |  |
| One parent Dutch | 36036 (5.6) | 692 (1.9) | 3578 (6.1) | 13668 (5.4) | 10058 (5.7) | 8040 (6.9) |
| Neither parent Dutch | 168019 (26.3) | 29721 (82.6) | 23843 (40.9) | 62926 (25.1) | 30213 (17.0) | 21316 (18.3) |
| Mother’s country of birth,  imputed = 22 (<0.01%) |  |  |  |  |  |  |
| Netherlands | 508979 (79.7) | 7297 (20.3) | 39404 (67.6) | 209405 (83.5) | 155623 (87.7) | 97250 (83.6) |
| Other European | 41241 (6.5) | 6542 (18.2) | 5016 (8.6) | 12132 (4.8) | 8046 (4.5) | 9505 (8.2) |
| Turkey | 8672 (1.4) | 2270 (6.3) | 1585 (2.7) | 3334 (1.3) | 1029 (0.6) | 454 (0.4) |
| Morocco | 12556 (2.0) | 3088 (8.6) | 2394 (4.1) | 4996 (2.0) | 1621 (0.9) | 457 (0.4) |
| Suriname | 4690 (0.7) | 227 (0.6) | 735 (1.3) | 2310 (0.9) | 907 (0.5) | 511 (0.4) |
| Dutch Caribbean | 4764 (0.7) | 235 (0.7) | 923 (1.6) | 2449 (1.0) | 740 (0.4) | 417 (0.4) |
| Indonesia | 1684 (0.3) | 162 (0.5) | 174 (0.3) | 537 (0.2) | 454 (0.3) | 357 (0.3) |
| Other African | 16314 (2.6) | 7117 (19.8) | 2536 (4.4) | 4266 (1.7) | 1390 (0.8) | 1005 (0.9) |
| Other Asian | 32092 (5.0) | 8436 (23.4) | 4586 (7.9) | 8937 (3.6) | 5832 (3.3) | 4301 (3.7) |
| American or Oceanian | 8015 (1.3) | 607 (1.7) | 945 (1.6) | 2548 (1.0) | 1892 (1.1) | 2023 (1.7) |
| Mother with insurance debt, yes,  imputed = 22 (<0.01%) | 18599 (2.9) | 2197 (6.1) | 6346 (10.9) | 9084 (3.6) | 798 (0.4) | 174 (0.1) |
| Mother was a criminal suspect, yes,  imputed =22 (<0.01%) | 6792 (1.1) | 1129 (3.1) | 2099 (3.6) | 2958 (1.2) | 461 (0.3) | 145 (0.1) |
| Mother’s social economic category,  imputed = 1282 (0.2%) |  |  |  |  |  |  |
| Employee | 396294 (62.0) | 4050 (11.3) | 16403 (28.1) | 148380 (59.1) | 135538 (76.3) | 91923 (79.1) |
| Director/majority shareholder | 2024 (0.3) | 24 (0.1) | 109 (0.2) | 498 (0.2) | 675 (0.4) | 718 (0.6) |
| Independent entrepreneur | 23751 (3.7) | 526 (1.5) | 1170 (2.0) | 7659 (3.1) | 7319 (4.1) | 7077 (6.1) |
| Other independent worker | 4644 (0.7) | 194 (0.5) | 570 (1.0) | 2333 (0.9) | 1040 (0.6) | 507 (0.4) |
| Receiving social assistance benefits | 786 (0.1) | 48 (0.1) | 120 (0.2) | 375 (0.1) | 157 (0.1) | 86 (0.1) |
| Receiving other social service benefits | 35681 (5.6) | 13133 (36.5) | 9035 (15.5) | 11447 (4.6) | 1588 (0.9) | 478 (0.4) |
| Receiving unemployment benefits | 27549 (4.3) | 2777 (7.7) | 5558 (9.5) | 11326 (4.5) | 5093 (2.9) | 2795 (2.4) |
| Receiving sickness benefits | 68489 (10.7) | 3822 (10.6) | 8668 (14.9) | 35038 (14.0) | 14489 (8.2) | 6472 (5.6) |
| Receiving pension | 185 (0.0) | 32 (0.1) | 27 (0.0) | 79 (0.0) | 36 (0.0) | 11 (0.0) |
| Preschooler/student with income | 8291 (1.3) | 322 (0.9) | 2474 (4.2) | 4841 (1.9) | 527 (0.3) | 127 (0.1) |
| Preschooler/student without income | 2563 (0.4) | 432 (1.2) | 508 (0.9) | 1054 (0.4) | 429 (0.2) | 140 (0.1) |
| Other without income | 66018 (10.3) | 10457 (29.1) | 13213 (22.7) | 26363 (10.5) | 10176 (5.7) | 5809 (5.0) |
| Cooperating family member | 2732 (0.4) | 164 (0.5) | 443 (0.8) | 1521 (0.6) | 467 (0.3) | 137 (0.1) |
| Household income percentile,  imputed = 10222 (1.6%) | - |  |  |  |  |  |
| <21^st^ percentile | 106031 (16.6) | 22834 (63.5) | 24564 (42.1) | 44972 (17.9) | 10040 (5.7) | 3621 (3.1) |
| 21^st^-40^th^ percentile | 76551 (12.0) | 5140 (14.3) | 11608 (19.9) | 41796 (16.7) | 13216 (7.4) | 4791 (4.1) |
| 41^st^ -60^th^ percentile | 113826 (17.8) | 3118 (8.7) | 9591 (16.5) | 60556 (24.1) | 30212 (17.0) | 10349 (8.9) |
| 61^st^-80^th^ percentile | 156395 (24.5) | 1796 (5.0) | 6585 (11.3) | 64163 (25.6) | 57560 (32.4) | 26291 (22.6) |
| >80^th^ percentile | 177314 (27.7) | 1058 (2.9) | 3676 (6.3) | 36184 (14.4) | 65642 (37.0) | 70754 (60.8) |
| Private household with unknown income | 3388 (0.5) | 634 (1.8) | 716 (1.2) | 1221 (0.5) | 482 (0.3) | 335 (0.3) |
| Institutional household | 2803 (0.4) | 1210 (3.4) | 754 (1.3) | 540 (0.2) | 205 (0.1) | 94 (0.1) |
| Private student household | 2699 (0.4) | 191 (0.5) | 804 (1.4) | 1482 (0.6) | 177 (0.1) | 45 (0.0) |
| Mother in same household as infant’s father, yes,  missing = 43845 (6.9%)* | 526718 (88.5) | 22729 (80.2) | 36500 (74.6) | 200839 (86.2) | 160244 (93.1) | 106406 (94.2) |
| Father’s educational level,  missing = 119651 (18.7%)* |  |  |  |  |  |  |
| Primary education | 25816 (5.0) | 8709 (39.8) | 5430 (12.9) | 8839 (4.4) | 2170 (1.4) | 668 (0.7) |
| Lower secondary education | 51063 (9.8) | 4537 (20.7) | 10150 (24.0) | 26611 (13.2) | 7583 (5.0) | 2182 (2.1) |
| Higher secondary education | 219194 (42.2) | 6501 (29.7) | 21421 (50.7) | 117573 (58.3) | 55437 (36.6) | 18262 (17.9) |
| Bachelor’s degree | 136126 (26.2) | 1438 (6.6) | 3953 (9.4) | 37559 (18.6) | 59617 (39.3) | 33559 (32.9) |
| Master’s or higher | 87157 (16.8) | 724 (3.3) | 1299 (3.1) | 10935 (5.4) | 26830 (17.7) | 47369 (46.4) |
| Vital outcome,  imputed = 27 (<0.01%) |  |  |  |  |  |  |
| Antepartum stillbirths | 1354 (0.21) | 144 (0.40) | 179 (0.31) | 540 (0.22) | 332 (0.19) | 159 (0.14) |
| Intrapartum stillbirths | 244 (0.04) | 28 (0.08) | 33 (0.06) | 95 (0.04) | 58 (0.03) | 30 (0.03) |
| Early neonatal death | 998 (0.16) | 96 (0.27) | 100 (0.17) | 439 (0.18) | 224 (0.13) | 139 (0.12) |
| Late neonatal death | 229 (0.04) | 17 (0.05) | 33 (0.06) | 109 (0.04) | 46 (0.03) | 24 (0.02) |
| Gestational age,  imputed = 3662 (0.6%) |  |  |  |  |  |  |
| Extremely preterm | 1792 (0.28) | 130 (0.36) | 229 (0.39) | 802 (0.32) | 396 (0.22) | 235 (0.20) |
| Very preterm | 3475 (0.55) | 239 (0.67) | 409 (0.70) | 1530 (0.61) | 838 (0.47) | 459 (0.40) |
| Moderate/late preterm | 28166 (4.42) | 1709 (4.77) | 3224 (5.55) | 11772 (4.70) | 7175 (4.05) | 4286 (3.69) |
| Postterm | 8699 (1.36) | 586 (1.64) | 542 (0.93) | 2727 (1.09) | 2686 (1.52) | 2158 (1.86) |
| Hoftiezer percentile,  imputed = 9014 (1.4%) |  |  |  |  |  |  |
| 0-10% (small-for-gestational-age) | 62329 (9.8) | 5243 (14.6) | 7809 (13.4) | 25381 (10.1) | 14362 (8.1) | 9534 (8.2) |
| 90-100% (large-for-gestational-age) | 64961 (10.2) | 2637 (7.4) | 4981 (8.6) | 25858 (10.3) | 19663 (11.1) | 11822 (10.2) |
| 5-minute Apgar score,  imputed = 1074 (0.2%) |  |  |  |  |  |  |
| 0-3 | 3334 (0.52) | 308 (0.86) | 381 (0.66) | 1346 (0.54) | 830 (0.47) | 469 (0.40) |
| 4-6 | 8640 (1.36) | 658 (1.84) | 906 (1.56) | 3531 (1.41) | 2228 (1.26) | 1317 (1.13) |
| NICU admission,  imputed = 3662 (0.6%) |  |  |  |  |  |  |
| Gestational age <32 weeks | 4153 (0.65) | 275 (0.77) | 507 (0.87) | 1855 (0.74) | 970 (0.55) | 546 (0.47) |
| Gestational age ≥32 weeks | 8602 (1.35) | 610 (1.70) | 910 (1.57) | 3457 (1.38) | 2201 (1.24) | 1424 (1.23) |
| Severe congenital anomaly | 2978 (0.47) | 256 (0.71) | 289 (0.50) | 1196 (0.48) | 771 (0.44) | 466 (0.40) |

All variables are reported as frequencies with percentages in brackets. The frequencies of imputed values are given below each variable name.

*Variable was not imputed, so the percentages are out of the cases which were not missing.

### Table S3: Composite outcome and vital outcome rates stratified by future maternal education (2016-2017)

| Outcome | Composite outcomes | | | Vital outcomes | | | | | |
| --- | --- | --- | --- | --- | --- | --- | --- | --- | --- |
| Outcome (sub)group | Any adverse outcome, % births | Any adverse outcome, % live births | Big 4 outcome, % live births | Stillbirths, /1000 births | Antepartum stillbirths, /1000 births | Intrapartum stillbirths, /1000 births | Total neonatal deaths, /1000 live births | Early neonatal deaths, /1000 live births | Late neonatal deaths, /1000 live births |
| Total population rate | 15.78 (15.65 to 15.91) | 15.57 (15.44 to 15.70) | 15.09 (14.96 to 15.22) | 2.46 (2.29 to 2.63) | 2.09 (1.93 to 2.25) | 0.36 (0.29 to 0.43) | 1.89 (1.74 to 2.04) | 1.53 (1.39 to 1.67) | 0.36 (0.29 to 0.43) |
| *Maternal education* |  |  |  |  |  |  |  |  |  |
| Primary education, rate | 21.39 (20.80 to 21.98) | 20.93 (20.35 to 21.51) | 20.39 (19.81 to 20.97) | 5.84 (4.74 to 6.94) | 4.71 (3.73 to 5.69) | 1.13 (0.65 to 1.61) | 2.94 (2.16 to 3.72) | 2.40 (1.70 to 3.10) | 0.53 (0.20 to 0.86) |
| Lower secondary, rate | 20.63 (20.01 to 21.25) | 20.39 (19.78 to 21.00) | 19.83 (19.22 to 20.44) | 2.97 (2.14 to 3.80) | 2.63 (1.85 to 3.41) | 0.34 (0.06 to 0.62) | 2.15 (1.44 to 2.86) | 1.58 (0.97 to 2.19) | 0.56 (0.20 to 0.92) |
| Higher secondary, rate | 16.67 (16.46 to 16.88) | 16.45 (16.25 to 16.65) | 15.99 (15.79 to 16.19) | 2.61 (2.33 to 2.89) | 2.22 (1.96 to 2.48) | 0.39 (0.28 to 0.50) | 2.12 (1.87 to 2.37) | 1.71 (1.48 to 1.94) | 0.41 (0.30 to 0.52) |
| Bachelor’s, rate | 13.89 (13.66 to 14.12) | 13.71 (13.48 to 13.94) | 13.23 (13.01 to 13.45) | 2.12 (1.82 to 2.42) | 1.76 (1.49 to 2.03) | 0.36 (0.24 to 0.48) | 1.58 (1.32 to 1.84) | 1.31 (1.07 to 1.55) | 0.27 (0.16 to 0.38) |
| Master’s or higher, rate (ref.) | 13.42 (13.14 to 13.70) | 13.28 (13.00 to 13.56) | 12.79 (12.52 to 13.06) | 1.63 (1.30 to 1.96) | 1.47 (1.16 to 1.78) | 0.16 (0.06 to 0.26) | 1.51 (1.19 to 1.83) | 1.25 (0.96 to 1.54) | 0.25 (0.12 to 0.38) |
| Rate difference | 7.97 (7.32 to 8.62) | 7.65 (7.00 to 8.30) | 7.60 (6.96 to 8.24) | 4.21 (3.07 to 5.35) | 3.24 (2.21 to 4.27) | 0.97 (0.48 to 1.46) | 1.43 (0.59 to 2.27) | 1.15 (0.39 to 1.91) | 0.28 (-0.07 to 0.63) |
| Rate ratio | 1.59 (1.54 to 1.64) | 1.58 (1.53 to 1.63) | 1.59 (1.53 to 1.65) | 3.59 (2.60 to 4.58) | 3.20 (2.25 to 4.15) | 7.20 (1.64 to 12.76) | 1.95 (1.29 to 2.61) | 1.91 (1.20 to 2.62) | 2.13 (0.41 to 3.85) |
| *Crude results* |  |  |  |  |  |  |  |  |  |
| PAR | 2.36 (2.05 to 2.67) | 2.29 (1.99 to 2.59) | 2.30 (2.00 to 2.60) | 0.83 (0.46 to 1.20) | 0.62 (0.27 to 0.97) | 0.21 (0.09 to 0.33) | 0.38 (0.03 to 0.73) | 0.28 (-0.04 to 0.60) | 0.11 (-0.03 to 0.25) |
| PAF, % | 14.9 (13.0 to 16.9) | 14.7 (12.8 to 16.7) | 15.2 (13.3 to 17.2) | 33.7 (18.4 to 49.0) | 29.6 (12.7 to 46.5) | 57.0 (22.1 to 92.0) | 20.2 (1.5 to 38.9) | 18.0 (-2.5 to 38.5) | 29.6 (-9.7 to 68.8) |
| Annual attributable cases | 3817 (3321 to 4313) | 3701 (3208 to 4194) | 3713 (3228 to 4198) | 134 (73 to 195) | 100 (42 to 158) | 34 (12 to 56) | 61 (4 to 118) | 44 (-6 to 94) | 17 (-6 to 40) |
| *Adjusted results* |  |  |  |  |  |  |  |  |  |
| PAR | 2.96 (2.65 to 3.27) | 2.90 (2.60 to 3.20) | 2.88 (2.58 to 3.18) | 0.87 (0.50 to 1.24) | 0.65 (0.30 to 1.00) | 0.22 (0.10 to 0.34) | 0.37 (0.02 to 0.72) | 0.26 (-0.06 to 0.58) | 0.11 (-0.03 to 0.25) |
| PAF, % | 18.8 (16.8 to 20.7) | 18.6 (16.6 to 20.6) | 19.1 (17.1 to 21.1) | 35.3 (20.0 to 50.6) | 31.2 (14.2 to 48.1) | 59.1 (24.4 to 93.8) | 19.5 (1.0 to 38.1) | 16.9 (-3.9 to 37.7) | 30.9 (-10.2 to 71.9) |
| Annual attributable cases | 4799 (4301 to 5297) | 4677 (4184 to 5171) | 4654 (4167 to 5141) | 140 (79 to 201) | 105 (47 to 163) | 35 (13 to 57) | 59 (3 to 115) | 42 (-10 to 94) | 18 (-6 to 42) |

The values provided are pooled means across all imputed datasets, with the pooled 95% confidence intervals given in brackets. Rate difference: rate in lowest education group minus rate in highest education group. Rate ratio: rate in lowest education group divided by rate in highest education group. Adjusted results are adjusted for age and parity. The Big 4 outcomes are preterm birth, small-for-gestational-age, Apgar <7, and severe congenital anomaly. PAF = population attributable fraction; PAR = population attributable risk.

### Table S4: Preterm birth and small-for-gestational-age rates stratified by future maternal education (2016-2017)

| Outcome | Preterm births | | | | Small-for-gestational age |
| --- | --- | --- | --- | --- | --- |
| Outcome (sub)group | Total PTBs (<37 weeks), % | Extremely PTBs (<28 weeks), % | Very PTBs (28-32 weeks), % | Moderate/late PTBs (33-36 weeks), % | Hoftiezer percentile <10, % |
| Total population rate | 5.15 (5.07 to 5.23) | 0.22 (0.20 to 0.24) | 0.52 (0.49 to 0.55) | 4.42 (4.35 to 4.49) | 9.70 (9.60 to 9.80) |
| *Maternal education* |  |  |  |  |  |
| Primary education, rate | 5.85 (5.51 to 6.19) | 0.29 (0.21 to 0.37) | 0.57 (0.46 to 0.68) | 4.98 (4.67 to 5.29) | 14.43 (13.92 to 14.94) |
| Lower secondary, rate | 6.47 (6.09 to 6.85) | 0.27 (0.19 to 0.35) | 0.72 (0.59 to 0.85) | 5.48 (5.13 to 5.83) | 13.64 (13.12 to 14.16) |
| Higher secondary, rate | 5.55 (5.42 to 5.68) | 0.26 (0.23 to 0.29) | 0.58 (0.54 to 0.62) | 4.71 (4.59 to 4.83) | 10.29 (10.12 to 10.46) |
| Bachelor’s, rate | 4.70 (4.56 to 4.84) | 0.17 (0.14 to 0.20) | 0.44 (0.40 to 0.48) | 4.09 (3.96 to 4.22) | 8.12 (7.94 to 8.30) |
| Master’s, rate (ref.) | 4.31 (4.14 to 4.48) | 0.15 (0.12 to 0.18) | 0.41 (0.36 to 0.46) | 3.75 (3.60 to 3.90) | 8.08 (7.86 to 8.30) |
| Rate difference | 1.54 (1.16 to 1.92) | 0.14 (0.06 to 0.22) | 0.17 (0.05 to 0.29) | 1.24 (0.89 to 1.59) | 6.34 (5.79 to 6.89) |
| Rate ratio | 1.36 (1.27 to 1.45) | 1.89 (1.25 to 2.53) | 1.41 (1.09 to 1.73) | 1.33 (1.23 to 1.43) | 1.78 (1.70 to 1.86) |
| *Crude results* |  |  |  |  |  |
| PAR | 0.85 (0.67 to 1.03) | 0.06 (0.02 to 0.10) | 0.11 (0.05 to 0.17) | 0.67 (0.50 to 0.84) | 1.62 (1.38 to 1.86) |
| PAF, % | 16.4 (12.9 to 20.0) | 29.8 (12.0 to 47.6) | 21.7 (10.3 to 33.2) | 15.2 (11.3 to 19.0) | 16.7 (14.2 to 19.2) |
| Annual attributable cases | 1367 (1072 to 1662) | 105 (42 to 168) | 182 (86 to 278) | 1080 (804 to 1356) | 2615 (2218 to 3012) |
| *Adjusted results* |  |  |  |  |  |
| PAR | 0.99 (0.81 to 1.17) | 0.06 (0.02 to 0.10) | 0.14 (0.08 to 0.20) | 0.79 (0.62 to 0.96) | 2.03 (1.79 to 2.27) |
| PAF, % | 19.2 (15.7 to 22.8) | 29.8 (12.0 to 47.6) | 26.8 (15.6 to 37.9) | 17.8 (14.0 to 21.7) | 21.0 (18.4 to 23.5) |
| Annual attributable cases | 1599 (1302 to 1896) | 104 (41 to 167) | 224 (130 to 318) | 1271 (995 to 1547) | 3284 (2885 to 3683) |

The values provided are pooled means across all imputed datasets, with the pooled 95% confidence intervals given in brackets. Rate difference: rate in lowest education group minus rate in highest education group. Rate ratio: rate in lowest education group divided by rate in highest education group. All rates are reported as the proportion of live births. Adjusted results are adjusted for age and parity. PAF = population attributable fraction; PAR = population attributable risk; PTB = preterm birth.

### Table S5: Apgar score <7, NICU admission, and severe congenital disease rates stratified by future maternal education (2016-2017)

| Outcome | 5-minute Apgar score | | | NICU admissions | | | Severe congenital diseases |
| --- | --- | --- | --- | --- | --- | --- | --- |
| Outcome (sub)group | Total Apgar scores <7, % | Apgar scores 0-3, % | Apgar scores 4-6, % | Total NICU admissions, % | <32 weeks NICU admissions, % | ≥32 weeks NICU admission, % | Severe congenital diseases, /1000 |
| Total population rate | 1.58 (1.54 to 1.62) | 0.28 (0.26 to 0.30) | 1.30 (1.26 to 1.34) | 1.98 (1.93 to 2.03) | 0.64 (0.61 to 0.67) | 1.34 (1.30 to 1.38) | 3.62 (3.41 to 3.83) |
| *Maternal education* |  |  |  |  |  |  |  |
| Primary education, rate | 2.32 (2.10 to 2.54) | 0.43 (0.34 to 0.52) | 1.88 (1.68 to 2.08) | 2.44 (2.22 to 2.66) | 0.75 (0.63 to 0.87) | 1.68 (1.50 to 1.86) | 5.34 (4.29 to 6.39) |
| Lower secondary, rate | 1.84 (1.63 to 2.05) | 0.31 (0.23 to 0.39) | 1.53 (1.34 to 1.72) | 2.57 (2.33 to 2.81) | 0.87 (0.73 to 1.01) | 1.70 (1.50 to 1.90) | 4.10 (3.12 to 5.08) |
| Higher secondary, rate | 1.64 (1.57 to 1.71) | 0.29 (0.26 to 0.32) | 1.36 (1.30 to 1.42) | 2.08 (2.00 to 2.16) | 0.75 (0.70 to 0.80) | 1.34 (1.28 to 1.40) | 3.73 (3.39 to 4.07) |
| Bachelor’s, rate | 1.47 (1.39 to 1.55) | 0.24 (0.21 to 0.27) | 1.23 (1.16 to 1.30) | 1.79 (1.70 to 1.88) | 0.53 (0.48 to 0.58) | 1.26 (1.19 to 1.33) | 3.45 (3.07 to 3.83) |
| Master’s or higher, rate (ref.) | 1.32 (1.23 to 1.41) | 0.26 (0.22 to 0.30) | 1.06 (0.98 to 1.14) | 1.72 (1.61 to 1.83) | 0.48 (0.42 to 0.54) | 1.24 (1.15 to 1.33) | 3.03 (2.58 to 3.48) |
| Rate difference | 0.99 (0.75 to 1.23) | 0.17 (0.07 to 0.27) | 0.82 (0.61 to 1.03) | 0.72 (0.47 to 0.97) | 0.28 (0.14 to 0.42) | 0.44 (0.23 to 0.65) | 2.31 (1.17 to 3.45) |
| Rate ratio | 1.75 (1.55 to 1.95) | 1.67 (1.22 to 2.12) | 1.77 (1.54 to 2.00) | 1.42 (1.26 to 1.58) | 1.58 (1.26 to 1.90) | 1.35 (1.17 to 1.53) | 1.76 (1.33 to 2.19) |
| *Crude results* |  |  |  |  |  |  |  |
| PAR | 0.26 (0.16 to 0.36) | 0.02 (-0.03 to 0.07) | 0.24 (0.15 to 0.33) | 0.27 (0.15 to 0.39) | 0.16 (0.10 to 0.22) | 0.10 (0.00 to 0.20) | 0.59 (0.10 to 1.08) |
| PAF, % | 16.2 (9.8 to 22.7) | 6.2 (-7.9 to 20.2) | 18.4 (11.3 to 25.5) | 13.4 (7.6 to 19.1) | 25.7 (15.6 to 35.9) | 7.5 (0.1 to 14.8) | 16.3 (2.6 to 30.0) |
| Annual attributable cases | 415 (250 to 580) | 28 (-36 to 92) | 387 (237 to 537) | 428 (243 to 613) | 266 (160 to 372) | 162 (2 to 322) | 95 (15 to 175) |
| *Adjusted results* |  |  |  |  |  |  |  |
| PAR | 0.35 (0.25 to 0.45) | 0.03 (-0.02 to 0.08) | 0.31 (0.22 to 0.40) | 0.38 (0.26 to 0.50) | 0.19 (0.13 to 0.25) | 0.19 (0.09 to 0.29) | 0.83 (0.34 to 1.32) |
| PAF, % | 21.9 (15.5 to 28.4) | 11.5 (-5.9 to 29.0) | 24.1 (16.9 to 31.4) | 19.0 (13.1 to 24.8) | 29.4 (19.6 to 39.2) | 14.0 (6.7 to 21.3) | 22.9 (9.2 to 36.6) |
| Annual attributable cases | 560 (394 to 726) | 52 (-27 to 131) | 508 (355 to 661) | 608 (420 to 796) | 304 (202 to 406) | 303 (145 to 461) | 134 (53 to 215) |

The values provided are pooled means across all imputed datasets, with the pooled 95% confidence intervals given in brackets. Rate difference: rate in lowest education group minus rate in highest education group. Rate ratio: rate in lowest education group divided by rate in highest education group. All rates are reported as the proportion of live births. Adjusted results are adjusted for age and parity. NICU = neonatal intensive care unit; PAF = population attributable fraction; PAR = population attributable risk.

### Table S6: Composite outcome and vital outcome rates stratified by maternal education in the unimputed dataset

| Outcome | Composite outcomes | | | Vital outcomes | | | | | |
| --- | --- | --- | --- | --- | --- | --- | --- | --- | --- |
| Outcome (sub)group | Any adverse outcome, % births | Any adverse outcome, % live births | Big 4 outcome, % live births | Stillbirths, /1000 births | Antepartum stillbirths, /1000 births | Intrapartum stillbirths, /1000 births | Total neonatal deaths, /1000 live births | Early neonatal deaths, /1000 live births | Late neonatal deaths, /1000 live births |
| Total population rate | 15.58 (15.49 to 15.67) | 15.38 (15.29 to 15.47) | 14.91 (14.82 to 15.00) | 2.40 (2.27 to 2.53) | 2.03 (1.91 to 2.15) | 0.37 (0.32 to 0.42) | 1.87 (1.76 to 1.98) | 1.49 (1.39 to 1.59) | 0.38 (0.33 to 0.43) |
| *Maternal education* |  |  |  |  |  |  |  |  |  |
| Primary education, rate | 21.04 (20.53 to 21.55) | 20.65 (20.14 to 21.16) | 20.08 (19.58 to 20.58) | 4.81 (3.94 to 5.68) | 3.94 (3.15 to 4.73) | 0.87 (0.50 to 1.24) | 3.46 (2.72 to 4.20) | 2.75 (2.09 to 3.41) | 0.71 (0.37 to 1.05) |
| Lower secondary, rate | 20.77 (20.41 to 21.13) | 20.47 (20.11 to 20.83) | 19.94 (19.58 to 20.30) | 3.71 (3.17 to 4.25) | 3.17 (2.67 to 3.67) | 0.54 (0.33 to 0.75) | 2.19 (1.77 to 2.61) | 1.62 (1.26 to 1.98) | 0.57 (0.36 to 0.78) |
| Higher secondary, rate | 16.39 (16.24 to 16.54) | 16.18 (16.03 to 16.33) | 15.71 (15.56 to 15.86) | 2.43 (2.23 to 2.63) | 2.08 (1.89 to 2.27) | 0.35 (0.27 to 0.43) | 2.15 (1.96 to 2.34) | 1.70 (1.53 to 1.87) | 0.45 (0.36 to 0.54) |
| Bachelor’s, rate | 13.62 (13.45 to 13.79) | 13.44 (13.27 to 13.61) | 13.00 (12.84 to 13.16) | 2.12 (1.90 to 2.34) | 1.80 (1.59 to 2.01) | 0.32 (0.23 to 0.41) | 1.46 (1.27 to 1.65) | 1.20 (1.03 to 1.37) | 0.26 (0.18 to 0.34) |
| Master’s or higher, rate (ref.) | 13.24 (13.04 to 13.44) | 13.09 (12.89 to 13.29) | 12.63 (12.43 to 12.83) | 1.63 (1.39 to 1.87) | 1.35 (1.13 to 1.57) | 0.28 (0.18 to 0.38) | 1.38 (1.16 to 1.60) | 1.11 (0.91 to 1.31) | 0.27 (0.17 to 0.37) |
| Rate difference | 7.80 (7.25 to 8.35) | 7.55 (7.00 to 8.10) | 7.46 (6.92 to 8.00) | 3.19 (2.29 to 4.09) | 2.59 (1.77 to 3.41) | 0.60 (0.22 to 0.98) | 2.08 (1.31 to 2.85) | 1.64 (0.95 to 2.33) | 0.44 (0.09 to 0.79) |
| Rate ratio | 1.59 (1.54 to 1.64) | 1.58 (1.53 to 1.63) | 1.59 (1.54 to 1.64) | 2.96 (2.27 to 3.65) | 2.92 (2.16 to 3.68) | 3.16 (1.39 to 4.93) | 2.50 (1.83 to 3.17) | 2.47 (1.73 to 3.21) | 2.66 (1.07 to 4.25) |
| *Crude results* |  |  |  |  |  |  |  |  |  |
| PAR | 2.17 (1.94 to 2.40) | 2.10 (1.88 to 2.32) | 2.11 (1.89 to 2.33) | 0.77 (0.50 to 1.04) | 0.68 (0.43 to 0.93) | 0.09 (-0.02 to 0.20) | 0.49 (0.24 to 0.74) | 0.37 (0.15 to 0.59) | 0.12 (0.01 to 0.23) |
| PAF, % | 13.9 (12.5 to 15.3) | 13.7 (12.2 to 15.2) | 14.1 (12.6 to 15.6) | 32.3 (20.6 to 43.9) | 33.6 (21.1 to 46.1) | 24.8 (-6.4 to 56.1) | 26.2 (12.7 to 39.6) | 25.1 (9.8 to 40.3) | 30.5 (1.9 to 59.1) |
| Annual attributable cases | 3027 (2712 to 3342) | 2935 (2621 to 3249) | 2937 (2629 to 3245) | 110 (70 to 150) | 97 (60 to 134) | 13 (-3 to 29) | 69 (33 to 105) | 53 (20 to 86) | 17 (1 to 33) |
| *Adjusted results* |  |  |  |  |  |  |  |  |  |
| PAR | 2.83 (2.60 to 3.06) | 2.76 (2.54 to 2.98) | 2.74 (2.52 to 2.96) | 0.86 (0.59 to 1.13) | 0.74 (0.49 to 0.99) | 0.11 (0.00 to 0.22) | 0.54 (0.29 to 0.79) | 0.40 (0.18 to 0.62) | 0.14 (0.03 to 0.25) |
| PAF, % | 18.2 (16.7 to 19.6) | 18.0 (16.5 to 19.4) | 18.4 (16.9 to 19.8) | 35.7 (24.1 to 47.2) | 36.6 (24.0 to 49.1) | 30.8 (-1.0 to 62.6) | 28.7 (15.3 to 42.2) | 26.6 (11.6 to 41.6) | 37.1 (7.2 to 67.0) |
| Annual attributable cases | 3952 (3636 to 4268) | 3848 (3534 to 4162) | 3816 (3507 to 4125) | 121 (81 to 161) | 105 (68 to 142) | 16 (-1 to 33) | 76 (40 to 112) | 56 (24 to 88) | 20 (4 to 36) |

The results provided are from the unimputed dataset where rows where missing values were excluded in a case-wise manner. The values in brackets are the 95% confidence intervals. Rate difference: rate in lowest education group minus rate in highest education group. Rate ratio: rate in lowest education group divided by rate in highest education group. The Big 4 outcomes are preterm birth, small-for-gestational-age, Apgar <7, and severe congenital anomaly. PAF = population attributable fraction; PAR = population attributable risk.

#### Table S7: Preterm birth and small-for-gestational-age rates stratified by maternal education in the unimputed dataset

| Outcome | Preterm births | | | | Small-for-gestational age |
| --- | --- | --- | --- | --- | --- |
| Outcome (sub)group | Total PTBs (<37 weeks), % | Extremely PTBs (<28 weeks), % | Very PTBs (28-32 weeks), % | Moderate/late PTBs (33-36 weeks), % | Hoftiezer percentile <10, % |
| Total population rate | 5.10 (5.04 to 5.16) | 0.21 (0.20 to 0.22) | 0.50 (0.48 to 0.52) | 4.39 (4.34 to 4.44) | 9.41 (9.33 to 9.49) |
| *Maternal education* |  |  |  |  |  |
| Primary education, rate | 5.74 (5.45 to 6.03) | 0.30 (0.23 to 0.37) | 0.63 (0.53 to 0.73) | 4.80 (4.53 to 5.07) | 14.12 (13.68 to 14.56) |
| Lower secondary, rate | 6.67 (6.45 to 6.89) | 0.31 (0.26 to 0.36) | 0.69 (0.62 to 0.76) | 5.67 (5.46 to 5.88) | 13.43 (13.12 to 13.74) |
| Higher secondary, rate | 5.48 (5.39 to 5.57) | 0.25 (0.23 to 0.27) | 0.56 (0.53 to 0.59) | 4.68 (4.59 to 4.77) | 9.91 (9.79 to 10.03) |
| Bachelor’s, rate | 4.62 (4.52 to 4.72) | 0.17 (0.15 to 0.19) | 0.44 (0.41 to 0.47) | 4.01 (3.91 to 4.11) | 7.81 (7.68 to 7.94) |
| Master’s or higher, rate (ref.) | 4.19 (4.07 to 4.31) | 0.15 (0.13 to 0.17) | 0.38 (0.34 to 0.42) | 3.66 (3.55 to 3.77) | 7.88 (7.72 to 8.04) |
| Rate difference | 1.55 (1.23 to 1.87) | 0.15 (0.08 to 0.22) | 0.25 (0.14 to 0.36) | 1.14 (0.85 to 1.43) | 6.24 (5.77 to 6.71) |
| Rate ratio | 1.37 (1.29 to 1.45) | 2.04 (1.47 to 2.61) | 1.67 (1.36 to 1.98) | 1.31 (1.23 to 1.39) | 1.79 (1.72 to 1.86) |
| *Crude results* |  |  |  |  |  |
| PAR | 0.90 (0.77 to 1.03) | 0.06 (0.03 to 0.09) | 0.12 (0.08 to 0.16) | 0.71 (0.58 to 0.84) | 1.42 (1.24 to 1.60) |
| PAF, % | 17.6 (14.9 to 20.2) | 29.9 (16.7 to 43.0) | 24.7 (16.2 to 33.3) | 16.1 (13.3 to 19.0) | 15.1 (13.2 to 17.0) |
| Annual attributable cases | 1260 (1072 to 1448) | 89 (49 to 129) | 175 (114 to 236) | 996 (820 to 1172) | 1980 (1729 to 2231) |
| *Adjusted results* |  |  |  |  |  |
| PAR | 1.04 (0.91 to 1.17) | 0.06 (0.03 to 0.09) | 0.14 (0.10 to 0.18) | 0.83 (0.70 to 0.96) | 1.88 (1.70 to 2.06) |
| PAF, % | 20.4 (17.7 to 23.0) | 29.8 (16.7 to 42.9) | 28.6 (20.1 to 37.1) | 18.9 (16.1 to 21.8) | 20.0 (18.0 to 21.9) |
| Annual attributable cases | 1460 (1271 to 1649) | 89 (49 to 129) | 203 (142 to 264) | 1168 (991 to 1345) | 2616 (2365 to 2867) |

The results provided are from the unimputed dataset where rows where missing values were excluded in a case-wise manner. The values in brackets are the 95% confidence intervals. Rate difference: rate in lowest education group minus rate in highest education group. Rate ratio: rate in lowest education group divided by rate in highest education group. All rates are reported as the proportion of live births. Adjusted results are adjusted for age and parity. PAF = population attributable fraction; PAR = population attributable risk; PTB = preterm birth.

### Table S8: Apgar score <7, NICU admission, and severe congenital disease rates stratified by maternal education in the unimputed dataset

| Outcome | 5-minute Apgar score | | | NICU admissions | | | Severe congenital diseases |
| --- | --- | --- | --- | --- | --- | --- | --- |
| Outcome (sub)group | Total Apgar scores <7, % | Apgar scores 0-3, % | Apgar scores 4-6, % | Total NICU admissions, % | <32 weeks NICU admissions, % | ≥32 weeks NICU admission, % | Severe congenital diseases, /1000 |
| Total population rate | 1.59 (1.56 to 1.62) | 0.26 (0.25 to 0.27) | 1.32 (1.29 to 1.35) | 1.96 (1.92 to 2.00) | 0.64 (0.62 to 0.66) | 1.32 (1.29 to 1.35) | 4.41 (4.24 to 4.58) |
| *Maternal education* |  |  |  |  |  |  |  |
| Primary education, rate | 2.26 (2.07 to 2.45) | 0.39 (0.31 to 0.47) | 1.87 (1.70 to 2.04) | 2.58 (2.38 to 2.78) | 0.83 (0.72 to 0.94) | 1.74 (1.58 to 1.90) | 6.50 (5.49 to 7.51) |
| Lower secondary, rate | 1.85 (1.73 to 1.97) | 0.30 (0.25 to 0.35) | 1.55 (1.44 to 1.66) | 2.45 (2.31 to 2.59) | 0.89 (0.81 to 0.97) | 1.56 (1.45 to 1.67) | 4.71 (4.10 to 5.32) |
| Higher secondary, rate | 1.66 (1.61 to 1.71) | 0.28 (0.26 to 0.30) | 1.38 (1.33 to 1.43) | 2.08 (2.02 to 2.14) | 0.72 (0.69 to 0.75) | 1.35 (1.30 to 1.40) | 4.61 (4.33 to 4.89) |
| Bachelor’s, rate | 1.46 (1.40 to 1.52) | 0.24 (0.22 to 0.26) | 1.23 (1.18 to 1.28) | 1.75 (1.69 to 1.81) | 0.54 (0.50 to 0.58) | 1.21 (1.16 to 1.26) | 4.15 (3.84 to 4.46) |
| Master’s or higher, rate (ref.) | 1.34 (1.27 to 1.41) | 0.23 (0.20 to 0.26) | 1.11 (1.05 to 1.17) | 1.66 (1.58 to 1.74) | 0.46 (0.42 to 0.50) | 1.20 (1.13 to 1.27) | 3.77 (3.40 to 4.14) |
| Rate difference | 0.92 (0.72 to 1.12) | 0.16 (0.08 to 0.24) | 0.76 (0.58 to 0.94) | 0.92 (0.71 to 1.13) | 0.37 (0.25 to 0.49) | 0.55 (0.37 to 0.73) | 2.73 (1.65 to 3.81) |
| Rate ratio | 1.68 (1.52 to 1.84) | 1.68 (1.28 to 2.08) | 1.68 (1.50 to 1.86) | 1.55 (1.41 to 1.69) | 1.80 (1.51 to 2.09) | 1.46 (1.30 to 1.62) | 1.72 (1.40 to 2.04) |
| *Crude results* |  |  |  |  |  |  |  |
| PAR | 0.24 (0.16 to 0.32) | 0.03 (0.00 to 0.06) | 0.21 (0.14 to 0.28) | 0.29 (0.20 to 0.38) | 0.17 (0.12 to 0.22) | 0.12 (0.05 to 0.19) | 0.64 (0.23 to 1.05) |
| PAF, % | 15.3 (10.4 to 20.2) | 11.6 (-0.7 to 23.9) | 16.0 (10.7 to 21.4) | 14.8 (10.5 to 19.2) | 27.3 (19.9 to 34.7) | 8.8 (3.5 to 14.1) | 14.4 (5.2 to 23.6) |
| Annual attributable cases | 343 (233 to 453) | 43 (-3 to 89) | 300 (200 to 400) | 408 (288 to 528) | 246 (179 to 313) | 163 (65 to 261) | 90 (32 to 148) |
| *Adjusted results* |  |  |  |  |  |  |  |
| PAR | 0.34 (0.26 to 0.42) | 0.05 (0.02 to 0.08) | 0.29 (0.22 to 0.36) | 0.39 (0.30 to 0.48) | 0.19 (0.14 to 0.24) | 0.20 (0.13 to 0.27) | 0.87 (0.46 to 1.28) |
| PAF, % | 21.4 (16.6 to 26.3) | 17.9 (6.5 to 29.4) | 22.1 (16.8 to 27.5) | 20.1 (15.7 to 24.5) | 29.9 (22.7 to 37.2) | 15.4 (9.8 to 20.9) | 19.7 (10.4 to 29.0) |
| Annual attributable cases | 480 (371 to 589) | 67 (24 to 110) | 413 (313 to 513) | 554 (432 to 676) | 269 (203 to 335) | 285 (182 to 388) | 123 (65 to 181) |

The results provided are from the unimputed dataset where rows where missing values were excluded in a case-wise manner. The values in brackets are the 95% confidence intervals. Rate difference: rate in lowest education group minus rate in highest education group. Rate ratio: rate in lowest education group divided by rate in highest education group. All rates are reported as the proportion of live births. Adjusted results are adjusted for age and parity. NICU = neonatal intensive care unit; PAF = population attributable fraction; PAR = population attributable risk.

### Table S9: PAR, PAF, and annual attributable cases adjusted for all covariates

| Outcome | Composite outcomes | | | Vital outcomes | | | | | |
| --- | --- | --- | --- | --- | --- | --- | --- | --- | --- |
| Outcome (sub)group | Any adverse outcome, % births | Any adverse outcome, % live births | Big 4 outcome, % live births | Stillbirths, /1000 births | Antepartum stillbirths, /1000 births | Intrapartum stillbirths, /1000 births | Total neonatal deaths, /1000 live births | Early neonatal deaths, /1000 live births | Late neonatal deaths, /1000 live births |
| PAR | 2.08 (1.86 to 2.30) | 2.03 (1.81 to 2.25) | 2.02 (1.81 to 2.23) | 0.73 (0.47 to 0.99) | 0.65 (0.41 to 0.89) | 0.08 (-0.02 to 0.18) | 0.30 (0.06 to 0.54) | 0.18 (-0.04 to 0.40) | 0.12 (0.02 to 0.22) |
| PAF, % | 13.2 (11.8 to 14.6) | 13.0 (11.6 to 14.4) | 13.3 (11.9 to 14.8) | 29.3 (18.6 to 39.9) | 30.6 (19.1 to 42.0) | 22.1 (-6.9 to 51.2) | 15.8 (3.1 to 28.5) | 11.8 (-2.8 to 26.3) | 33.3 (6.4 to 60.1) |
| Annual attributable cases | 3328 (2981 to 3675) | 3234 (2890 to 3578) | 3213 (2875 to 3551) | 117 (74 to 160) | 103 (64 to 142) | 13 (-4 to 30) | 48 (9 to 87) | 29 (-7 to 65) | 19 (3 to 35) |

The values provided are pooled means across all imputed datasets, with the pooled 95% confidence intervals given in brackets. Results are adjusted for age, parity, province, mother’s migration background, mother’s parents’ nationalities, mother’s country of birth, mother with insurance debt, mother was a criminal suspect, mother’s occupational status, and household income percentile. The Big 4 outcomes are preterm birth, small-for-gestational-age, APGAR <7, and severe congenital anomaly. PAF = population attributable fraction; PAR = population attributable risk.

### Table S10: Preterm birth and small-for-gestational-age, PAR, PAF, and annual attributable cases adjusted for all covariates

| Outcome | Preterm births | | | | Small-for-gestational age |
| --- | --- | --- | --- | --- | --- |
| Outcome (sub)group | Total PTBs (<37 weeks), % | Extremely PTBs (<28 weeks), % | Very PTBs (28-32 weeks), % | Moderate/late PTBs (33-36 weeks), % | Hoftiezer percentile <10, % |
| Total population rate | 5.09 (5.04 to 5.14) | 0.22 (0.21 to 0.23) | 0.51 (0.49 to 0.53) | 4.36 (4.31 to 4.41) | 9.67 (9.60 to 9.74) |
| *Maternal education* |  |  |  |  |  |
| Primary education, rate | 5.51 (5.27 to 5.75) | 0.27 (0.22 to 0.32) | 0.60 (0.52 to 0.68) | 4.64 (4.42 to 4.86) | 14.42 (14.06 to 14.78) |
| Lower secondary, rate | 6.40 (6.17 to 6.63) | 0.32 (0.27 to 0.37) | 0.65 (0.58 to 0.72) | 5.43 (5.22 to 5.64) | 13.28 (12.96 to 13.60) |
| Higher secondary, rate | 5.47 (5.38 to 5.56) | 0.26 (0.24 to 0.28) | 0.57 (0.54 to 0.60) | 4.64 (4.56 to 4.72) | 10.03 (9.91 to 10.15) |
| Bachelor’s, rate | 4.62 (4.52 to 4.72) | 0.17 (0.15 to 0.19) | 0.44 (0.41 to 0.47) | 4.00 (3.91 to 4.09) | 8.03 (7.90 to 8.16) |
| Master’s or higher, rate (ref.) | 4.19 (4.07 to 4.31) | 0.16 (0.14 to 0.18) | 0.38 (0.34 to 0.42) | 3.65 (3.54 to 3.76) | 8.15 (7.99 to 8.31) |
| Rate difference | 1.33 (1.07 to 1.59) | 0.12 (0.06 to 0.18) | 0.22 (0.13 to 0.31) | 0.99 (0.75 to 1.23) | 6.27 (5.87 to 6.67) |
| Rate ratio | 1.32 (1.25 to 1.39) | 1.74 (1.31 to 2.17) | 1.58 (1.32 to 1.84) | 1.27 (1.20 to 1.34) | 1.77 (1.71 to 1.83) |
| *Fully adjusted results* |  |  |  |  |  |
| PAR | 1.02 (0.89 to 1.15) | 0.05 (0.02 to 0.08) | 0.14 (0.10 to 0.18) | 0.83 (0.71 to 0.95) | 1.14 (0.97 to 1.31) |
| PAF, % | 20.1 (17.5 to 22.6) | 23.3 (11.3 to 35.4) | 27.1 (19.4 to 34.8) | 19.1 (16.3 to 21.8) | 11.8 (10.0 to 13.6) |
| Annual attributable cases | 1626 (1421 to 1831) | 82 (39 to 125) | 220 (157 to 283) | 1323 (1132 to 1514) | 1823 (1545 to 2101) |

The values provided are pooled means across all imputed datasets, with the pooled 95% confidence intervals given in brackets. Rate difference: rate in lowest education group minus rate in highest education group. Rate ratio: rate in lowest education group divided by rate in highest education group. All rates are reported as the proportion of live births. Fully adjusted results are adjusted for age, parity, province, mother’s migration background, mother’s parents’ nationalities, mother’s country of birth, mother with insurance debt, mother was a criminal suspect, mother’s occupational status, and household income percentile. PAF = population attributable fraction; PAR = population attributable risk; PTB = preterm birth.

### Table S11: Apgar score <7, NICU admission, and severe congenital disease, PAR, PAF, and annual attributable cases adjusted for all covariates

| Outcome | 5-minute APGAR score | | | NICU admissions | | | Severe congenital diseases |
| --- | --- | --- | --- | --- | --- | --- | --- |
| Outcome (sub)group | Total APGAR scores <7, % | APGAR scores 0-3, % | APGAR scores 4-6, % | Total NICU admissions, % | <32 weeks NICU admissions, % | ≥32 weeks NICU admission, % | Severe congenital diseases, /1000 |
| Total population rate | 1.63 (1.60 to 1.66) | 0.27 (0.26 to 0.28) | 1.36 (1.33 to 1.39) | 2.00 (1.97 to 2.03) | 0.65 (0.63 to 0.67) | 1.35 (1.32 to 1.38) | 4.54 (4.37 to 4.71) |
| *Maternal education* |  |  |  |  |  |  |  |
| Primary education, rate | 2.22 (2.07 to 2.37) | 0.38 (0.32 to 0.44) | 1.84 (1.70 to 1.98) | 2.47 (2.31 to 2.63) | 0.77 (0.68 to 0.86) | 1.70 (1.57 to 1.83) | 6.81 (5.96 to 7.66) |
| Lower secondary, rate | 1.85 (1.72 to 1.98) | 0.29 (0.24 to 0.34) | 1.56 (1.44 to 1.68) | 2.44 (2.30 to 2.58) | 0.87 (0.78 to 0.96) | 1.57 (1.45 to 1.69) | 4.75 (4.11 to 5.39) |
| Higher secondary, rate | 1.69 (1.64 to 1.74) | 0.28 (0.26 to 0.30) | 1.41 (1.36 to 1.46) | 2.12 (2.06 to 2.18) | 0.74 (0.71 to 0.77) | 1.38 (1.33 to 1.43) | 4.66 (4.39 to 4.93) |
| Bachelor’s, rate | 1.51 (1.45 to 1.57) | 0.25 (0.23 to 0.27) | 1.26 (1.21 to 1.31) | 1.79 (1.73 to 1.85) | 0.55 (0.52 to 0.58) | 1.24 (1.19 to 1.29) | 4.25 (3.95 to 4.55) |
| Master’s or higher, rate (ref.) | 1.38 (1.31 to 1.45) | 0.24 (0.21 to 0.27) | 1.13 (1.07 to 1.19) | 1.70 (1.63 to 1.77) | 0.47 (0.43 to 0.51) | 1.23 (1.17 to 1.29) | 3.91 (3.55 to 4.27) |
| Rate difference | 0.84 (0.67 to 1.01) | 0.14 (0.07 to 0.21) | 0.70 (0.55 to 0.85) | 0.77 (0.59 to 0.95) | 0.30 (0.20 to 0.40) | 0.48 (0.33 to 0.63) | 2.90 (1.98 to 3.82) |
| Rate ratio | 1.61 (1.47 to 1.75) | 1.57 (1.25 to 1.89) | 1.62 (1.47 to 1.77) | 1.46 (1.35 to 1.57) | 1.63 (1.40 to 1.86) | 1.39 (1.26 to 1.52) | 1.74 (1.47 to 2.01) |
| *Fully adjusted results* |  |  |  |  |  |  |  |
| PAR | 0.26 (0.19 to 0.33) | 0.03 (0.00 to 0.06) | 0.23 (0.16 to 0.30) | 0.36 (0.28 to 0.44) | 0.18 (0.14 to 0.22) | 0.18 (0.11 to 0.25) | 0.50 (0.10 to 0.90) |
| PAF, % | 15.7 (11.2 to 20.2) | 10.3 (-0.3 to 21.0) | 16.8 (11.9 to 21.7) | 18.0 (13.9 to 22.1) | 27.4 (20.6 to 34.1) | 13.5 (8.3 to 18.7) | 11.1 (2.3 to 19.9) |
| Annual attributable cases | 407 (291 to 523) | 45 (-1 to 91) | 362 (256 to 468) | 574 (442 to 706) | 284 (213 to 355) | 290 (178 to 402) | 80 (17 to 143) |

The values provided are pooled means across all imputed datasets, with the pooled 95% confidence intervals given in brackets. Rate difference: rate in lowest education group minus rate in highest education group. Rate ratio: rate in lowest education group divided by rate in highest education group. All rates are reported as the proportion of live births. Fully adjusted results are adjusted for age, parity, province, mother’s migration background, mother’s parents’ nationalities, mother’s country of birth, mother with insurance debt, mother was a criminal suspect, mother’s occupational status, and household income percentile. NICU = neonatal intensive care unit; PAF = population attributable fraction; PAR = population attributable risk.
